# Niche partitioning of microbial communities in riverine floodplains

**DOI:** 10.1038/s41598-019-52865-4

**Published:** 2019-11-08

**Authors:** Marc Peipoch, Scott R. Miller, Tiago R. Antao, H. Maurice Valett

**Affiliations:** 1 Stroud Water Research Center, Avondale, PA USA; 2 Division of Biological Sciences, University of Montana, Missoula, MT USA

**Keywords:** Freshwater ecology, Element cycles, Biodiversity

## Abstract

Riverine floodplains exhibit high floral and faunal diversity as a consequence of their biophysical complexity. Extension of such niche partitioning processes to microbial communities is far less resolved or supported. Here, we evaluated the responses of aquatic biofilms diversity to environmental gradients across ten riverine floodplains with differing degrees of flow alteration and habitat diversity to assess whether complex floodplains support biofilm communities with greater biodiversity and species interactions. No significant evidence was found to support a central role for habitat diversity in promoting microbial diversity across 116 samples derived from 62 aquatic habitats, as neither α (H’: 2.8–4.1) nor β (Sørensen: 0.3–0.39) diversity were positively related to floodplain complexity across the ten floodplains. In contrast, our results documented the sensitivity of biofilm communities to regional templates manifested as gradients of carbon, nitrogen, and phosphorous availability. Large-scale conditions reflecting nitrogen limitation increased the relative abundance of N-fixing cyanobacteria (up to 0.34 as fraction of total reads), constrained the total number of interactions among bacterial taxa, and reinforced negative over positive interactions, generating unique microbial communities and networks that reflect large-scale species sorting in response to regional geochemical gradients.

## Introduction

Unraveling the biogeochemistry^1–3^, trophic significance^4–6^, and biodiversity^7,8^ of microbial assemblages attached to hard substrata of riverbeds–aka epilithic biofilms–has been a dominant focus of aquatic ecology, leading to recognition of epilithic biofilms as key control agents of ecosystem processes. The structure of river biofilms have been examined at several spatial scales, from species segregation within a few nanometers^9,10^ to differentiation of biofilm functions along river sections^11,12^, and landscape-scale responses of biotic constituents to changes in ecosystem integrity^13^. Inasmuch as biofilm assemblages are becoming increasingly scrutinized at multiple scales and scenarios, little is known about the assembly of biofilm communities among and within riverine floodplains, one of the most iconic elements of fluvial landscapes.

Adjacent to streams and rivers of different sizes, well-preserved floodplains are the paradigm of landscape heterogeneity, blending terrestrial and aquatic habitats as a mosaic of patches characterized by high turnover rates^14–16^. Habitat complexity of this kind gives rise to remarkable gradients of environmental conditions and resource availability across multiple spatial scales^17,18^. Complex floodplains are carved by cut-and-fill alluviation that sustains a variety of ecological niches and biological species, making them one of the world’s most biologically diverse ecosystems in relation to their area^15,19^. Floodplain complexity has been shown to enhance biodiversity of plants^20^, invertebrates^21,22^, fish^23,24^ and birds^25^. Some have argued that river floodplains are the “primary arena” for species interactions across vast regional landscapes^26^. This perspective relies on a direct association between niche heterogeneity and species diversity as a fundamental mechanism explaining enhanced species richness and distribution across riverine floodplains, a relationship that has been conceptualized and tested only for organisms of much larger size than microbes.

Whether it is appropriate to transfer ecological theory developed from and for macro-organisms (*i*.*e*., those visible to the naked eye) to the ecology of microbial communities is an ongoing debate^27,28^. Differences in dispersal capabilities and rates of local adaptation are undoubtedly to be given weight when rationalizing how micro- and macro-organisms may respond to habitat complexity and environmental gradients presented by riverine floodplains (*i*.*e*., niche partitioning). For instance, hydrologic regimes are known to exert powerful controls on invertebrate^29,30^ and fish^31^ communities by favoring specific lifecycles, but large-scale interactions between hydrology and biofilm communities are far less predictable and less well understood^32^. As the ecotone between terrestrial and aquatic landscape elements, floodplains include different ‘zones’ of organization. Main channel habitats (*e*.*g*., riffles, pools, shallow shorelines) occur within a zone of transport and connectivity linking upstream and downstream segments^16,33^. Off-channel habitats include parafluvial elements residing near and frequently disturbed by flowing water and orthofluvial patches with vegetation development characteristic of later stages of succession due to infrequent exposure to flooding disturbance. Springbrooks, side channels, and ponds represent a diverse array of niches in the off-channel zone. While these floodplain habitats include a plethora of environmental opportunities^34,35^, such habitat heterogeneity occurs over hundreds (if not thousands) of meters and may last several days or weeks. Considering the great dispersal capabilities and rapid rates of adaptation characteristic of microbial communities like epilithic biofilms, the extent to which biofilm assemblages reflect and respond to environmental heterogeneity occurring at these spatial and temporal scales is unclear. Hence, it remains to be seen how variation in the degree of habitat and environmental heterogeneity among complex floodplains translates into more ‘complex’ benthic biofilm communities with greater numbers of microbial taxa and species interactions.

Despite uncertainties surrounding the general sensitivity of microbial diversity to environmental gradients^36^, biogeochemical controls on river biofilms have been identified at multiple levels of ecological organization including microhabitats^37^, stream reaches^38^, and watersheds^13^, all with significant effects on the microbial diversity of biofilm communities. More recently, a growing body of research has begun to incorporate network analysis based on microbial co-occurrence patterns (*i*.*e*., microbial association networks) to better understand interactions among microbial taxa, community-level responses to environmental conditions, and to elucidate keystone taxa in microbial communities^13,28,39^. Network analysis is a powerful approach for identifying spatial associations among microbial taxa, especially when communities are assessed across pronounced environmental gradients to which different taxa may respond in similar or opposite manners. Some authors have argued that network analysis is more suitable than diversity metrics (*e*.*g*., alpha or beta diversity) when dealing with complex microbial datasets^40^. Interpreting microbial interactions and identifying keystone taxa by exploring microbial association networks may be particularly well suited for riverine floodplains given the degree of habitat heterogeneity that floodplains convey within small areas. Concomitantly, these approaches allow investigation of hierarchical controls over spatial patterns of diversity presented by the occurrence of a variety of floodplains within river networks draining diverse elements of regional landscapes.

Here, we compare environmental heterogeneity, microbial diversity, and co-occurrence microbial networks across riverine floodplains with differing flow regimes and habitat diversity. Ten riverine floodplains were sampled using a hierarchical approach to assess how biofilm assemblages respond to environmental gradients at biome, floodplain, zone, and habitat scales. Our goals were to 1) determine the extent to which complex riverine floodplains with greater habitat heterogeneity and more pronounced environmental gradients support biofilm communities with greater biodiversity and more extensive biological interaction (*i*.*e*., well connected biofilm networks), 2) document the occurrence of keystone taxa among epilithic assemblages, and 3) identify and characterize critical scales or organization for biofilm communities in riverine floodplains.

## Results

### Floodplain locations and river flow

**Figure 1:**
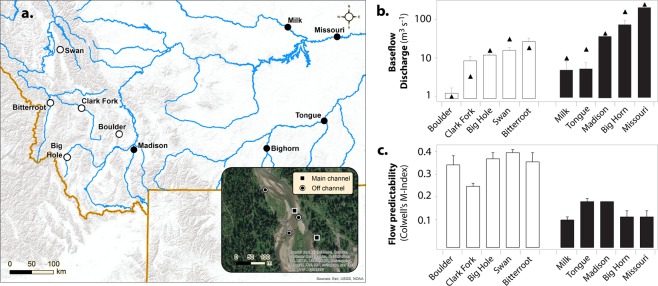
River location and flow. Map of Montana, USA, showing floodplain locations and pictorial representation of main- and off-channel sampling locations on the Swan River (**a**). Average baseflow discharge (m3/s) for the 1970-2017 period; triangles indicate river discharge during sampling; white bars correspond to floodplain locations in relatively free-flowing rivers, black bars correspond to river floodplains located downstream of one or more impoundments (**b**). Index of the predictability of flooding events in each river floodplain, color coding as in panel b, error bars were generated from 999 bootstrapped samples (**c**). Satellite imagery were obtained from World Imagery (http://goto.arcgisonline.com/maps/World_Imagery) and modified using ArcGIS 10.3 (https://www.esri.com/en-us/arcgis/products/arcgis-pro/overview). Credits attribution for both satellite and terrain imagery are Esri, DigitalGlobe, GeoEye, Earthstar Geographics, CNES/Airbus DS, USDA, USGS, AeroGRID, IGN, and the GIS User Community.

We selected ten riverine floodplains distributed across montane (n = 6) and grassland (n = 4) biomes within the Rocky Mountains and Great Plains of Montana, USA (Fig. 1a). Average baseflow discharge ranged from 1 to > 100 m^3^s^−1^ across rivers and biomes (Fig. 1b). Half of the floodplains, including all grassland systems, are located downstream of large scale impoundments, while the other half, mostly associated with montane rivers, are in relatively free-flowing waters (Fig. 1a,b). While extensive examination of flow regimes is beyond the scope of this study, Colwell’s M-Index^41^ was used to describe the effects of impoundments on flow seasonality. The index is minimal when the probabilities of occurrence for river flows of a given magnitude are independent of the season, *e*.*g*., less predictable flooding seasonality. Flow predictability was significantly lower with river dams present (0.11 ± 0.01) than when absent (0.27 ± 0.02; Fig. 1c). Long-term discharge data in free-flowing rivers show recurrent spring, snowmelt-driven floods characteristic of western USA, contrasting with more irregular seasonality in river flow for those floodplains under the influence of upstream dams (Fig. S1).

### Environmental variation across riverine floodplains

**Figure 2:**
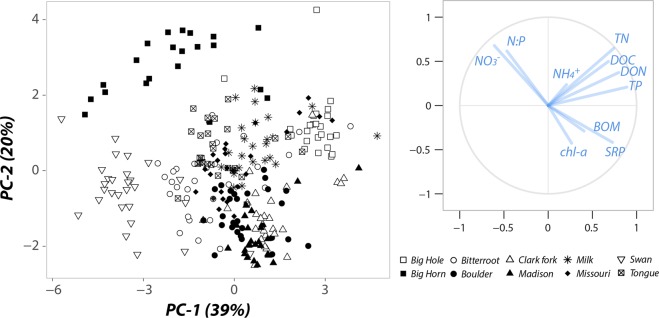
Environmental variation in riverine floodplains. Principal Components Analysis (PCA) of ten biogeochemical variables characterizing environmental heterogeneity for all habitats within each of the ten riverine floodplains. Left panel shows all PCA-scores (n = 265 habitats) along PCA-1 and -2 components. Unique symbol shapes represent individual floodplains. Right panel indicates the PCA loadings (biogeochemical variables) corresponding to each of the ten biogeochemical parameters measured in each sampled habitat.

Most of the observed variation in water chemistry was attributable to differences among individual floodplains rather than at scales above or within them (Table 1). Average concentrations of total nitrogen (TN) were greatest in the Bighorn, Missouri, and Milk Rivers (Table S1), where flow regime is also more heavily altered (Fig. 1c). The Big Hole River had the greatest concentrations of dissolved organic forms of carbon (C) and nitrogen (N), while the Swan River showed minimum concentrations for all chemical constituents except nitrate (N-NO_3_^−^; Table S1). Similar conditions of high phosphorus (P) availability and low N-NO_3_^−^ concentrations in the Clark Fork, Boulder, and Madison Rivers, suggest potential N-limitation of biological activity in aquatic habitats of these floodplains (Table S1). Variation in both biofilm biomass and chlorophyll-a abundance was mostly unexplained (*i*.*e*., residual variance 73 and 79%, respectively, Table 1), but variance component analysis revealed meaningful influence at the habitat-scale (Table 1). Ordination analysis separated riverine floodplains along its first component (PC-1) following a gradient of dissolved organic carbon (DOC) and total nutrient concentrations (Fig. 2). In contrast, PC-2 captured variation in bioavailable N, from high-NO_3_ waters (Bighorn, Big Hole, or Swan) to more N-limited floodplains with high P concentrations and greater chlorophyll-a abundance (Clark Fork, Boulder, Madison, Bitterroot; Fig. 2 and Table S1).

**Table 1:** Variance Component Analysis.

Parameter	Biome	Floodplain	Zone	Habitat	Residual
Dissolved Organic Carbon	0	63	4	13	20
Ammonium	0	0	13	0	87
Nitrate + Nitrite	0	60	4	2	34
Dissolved Organic Nitrogen	0	64	3	3	30
Total Nitrogen	11	63	9	0	17
Soluble Reactive Phosphorous	0	73	0	21	6
Total Phosphorous	0	87	1	9	4
molar N:P ratio	0	65	7	3	25
Benthic chlorophyll-a	0	8	0	13	79
Benthic organic matter	0	0	8	19	73

Values represent the estimated percentage of variance explained by each of the spatial scales and for each biogeochemical variable. Residual variance represents unexplained variation.

Biogeochemical variation within each floodplain (*i*.*e*., at zone and habitat scales) was widely spread across PC-1 and PC2 components depending on the floodplain (Fig. 2), which is in agreement with a substantial percentage of variation in DOC, P, chlorophyll-a abundance, and BOM attributed to the habitat scale (Table 1). Among habitats, highest concentrations of inorganic forms of N and P were observed in slow-moving waters (*e*.*g*., springbrooks) whereas TN and DON concentrations were significantly greater in floodplain ponds (Table S1). Overall, flow predictability (Colwell’s M-Index) was positively related to both habitat diversity (Shannon’s H, R = 0.74, Fig. S2-a) and environmental heterogeneity (CV for habitat centroids, R = 0.88, Fig. S2-b) across the ten riverine floodplains, indicating that floodplains in relatively free-flowing rivers should provide greater niche diversity for biofilm communities.

### Biofilm diversity and composition across riverine floodplains

Bacterial diversity (α-diversity as Shannon’s H index) varied significantly among floodplains (*F*_(8,107)_ = 5.07; *P* < 0.001) but not among biomes, zones, or habitat types (Table S2). Shannon’s index ranged from 2.8 in the Missouri River (503 bacterial families) to 4.1 in the Tongue River (660 bacterial families [Table S2]). Significant differences in β-diversity were also observed among floodplains (*R*^2^ = 0.2; *F*_(8,686)_ = 18.67; *P* < 0.001) but not at any other spatial scale (Table S2). At the floodplain level, most of the β-diversity reflected species replacement among habitats (β_turnover_) rather than a nested loss of bacterial taxa from rich to poor habitats (*i*.*e*., β_nestedness_ [Table S2]). Species turnover in biofilm communities across floodplains was dominated by increased relative abundance of *cyanobacteria* within select floodplains (up to 34% of total reads), with a 3-fold increase in cyanobacteria contributions to β-diversity in N-limited floodplains (average total of 33 ± 5%) compared to in N-rich floodplains (9 ± 4%). Differences in chlorophyll-a abundance among floodplains (Fig. 2) reflected variation in the relative abundances of both cyanobacteria and eukaryotic algae (Fig. S3-a). Eukaryotic algal composition varied among rivers (Fig. S3-b), but was dominated by photosynthetic stramenopiles (*i*.*e*., heterokont algae). Diatoms comprised 87% of the stramenopile sequence reads for the full data set and were the most abundant members of this group in most rivers (Fig. S3-c), although other stramenopiles (*e*.*g*., brown algae) were important contributors to the diversity of specific floodplains.

**Figure 3:**
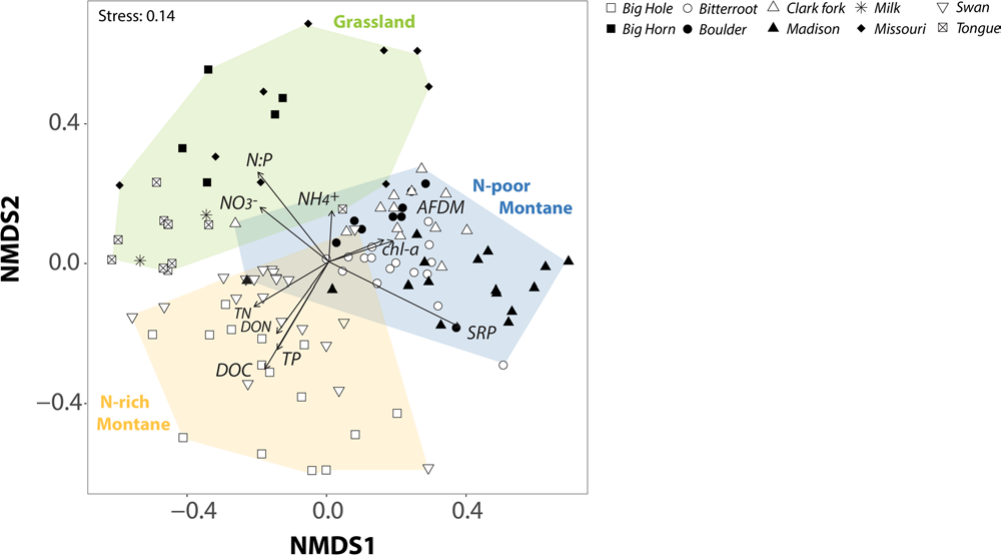
Bacterial community composition among floodplains. Non-metric Multidimensional Scaling (NMDS) based on Bray-Curtis dissimilarity. Data points correspond to biofilm samples (shape coding as in Fig. 2). Vectors correspond to projected environmental variables by finding directions in NMDS space with maximal correlation to each environmental variable. Polygons illustrate the grassland biome (green) and the two montane sub-biomes, N-rich (orange) and N-limited floodplains (blue). Statistical differences among clusters are detailed in Table S2.

**Figure 4:**
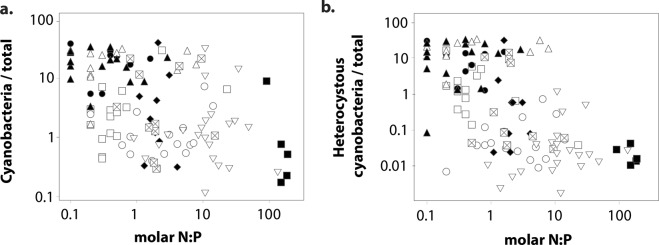
Relative abundance of cyanobacteria versus N:P. Sequence reads of (**a**) cyanobacteria and (**b**) heterocystous cyanobacteria are expressed as a fraction of total sequence reads. Data points correspond to biofilm samples (shape coding as in Fig. 2).

Bacterial community composition varied significantly between grassland and montane biomes (ANOSIM-R: 0.279) and more so across floodplains (ANOSIM-R: 0.679). Unconstrained ordination revealed distinct communities at more N-limited floodplains (Clark Fork, Madison, and Boulder), significantly different from those of other montane rivers or in grassland floodplains (Fig. 3; Table S3). As such, bacterial communities in montane floodplains separated in two distinct groups along a gradient of DOC, N, and P availability, from high NO_3_ and DOC concentrations in Swan and Big Hole Rivers (hereafter referred to as N-rich conditions) to more N-limited floodplains with greater P availability and chlorophyll-a abundance (Fig. 3; Table S1). The second NMDS axis separated bacterial communities between grassland floodplains and the two groups in the montane biome (Fig. 3). Together, these results suggest the occurrence of three sub-biomes experienced by epilithic biofilms of the rivers that flow within these regional geochemical gradients. Significant differences in both environmental conditions (ANOSIM-R: 0.215–0.415, *p*-value < 0.01) and bacterial community structure (ANOSIM-R: 0.215-0.687, Table S3) were observed among the three sub-biomes supporting the patterns observed in NMDS ordination. Community differentiation of a N-poor montane sub-biome from N-rich montane floodplains and the grassland biome is likely related to the increasing relative abundance of cyanobacteria in biofilm communities as the N:P ratio of the waters in which they reside declines (R = −0.46, *p*-value = 0.018, Fig. 4a). On average, N:P ratios in the N-poor sub-biome were an order of magnitude lower (1.8 ± 0.4) than in the N-rich sub-biome (12.7 ± 3.9) and grassland biome (30.7 ± 11.8). Variation in the relative abundance of a clade of multicellular cyanobacteria that produce specialized nitrogen-fixing cells (heterocysts) when N is limiting was also largely related to the floodplain in which they occurred (*R*^2^ = 0.71; *F*_(8,105)_ = 31.92; *P* < 0.0001). This pattern principally reflects differences in N:P ratios among rivers, since the proportion of heterocystous cyanobacterial reads decreased with increasing N:P ratios (*R* = −0.63, *p*-value = 0.009, Fig. 4b;). Only marginal differentiation of biofilm communities was observed between zones (ANOSIM-R: 0.051) and not at all among habitats (P > 0.05). Low variation in bacterial community similarity within floodplains concur with greater variation in water chemistry among floodplains than within them (Table 1), including the most significant predictors of variation in bacterial community similarity.

### Environmental controls on biofilm communities

Constrained ordination showed that concentrations of DOC and P (as soluble reactive phosphorous [SRP]) were the most significant predictors of variation in bacterial community similarity, with each accounting for a similar portion of the total variation explained by the model (Table S4-a). Phosphorous concentration was significantly correlated to the relative abundance of cyanobacteria across floodplain habitats (R = 0.58, P < 0.05), and this may be an avenue by which P abundance influenced overall differences in bacterial community composition. Total N and N:P ratios were also identified by the model as significant predictors, but with much lower proportions of variation in community structure attributed to them (Table S4-a). Once explanatory variables were selected, results of variance partitioning showed that geographic location was as good a predictor of bacterial community structure as the four selected environmental predictors combined, and the largest portion of the explained variation in community structure was ascribed to shared variation between geographic and environmental components (Table S4-b). Together, unconstrained and constrained ordination indicated that a combination of geographic location and biogeochemical condition accounted for the greatest amount of the variation in bacterial community structure at the habitat level (Table S4-b), although most variation remained unexplained (77.6%, Table S4-b).

### Co-occurrence bacterial networks

**Figure 5:**
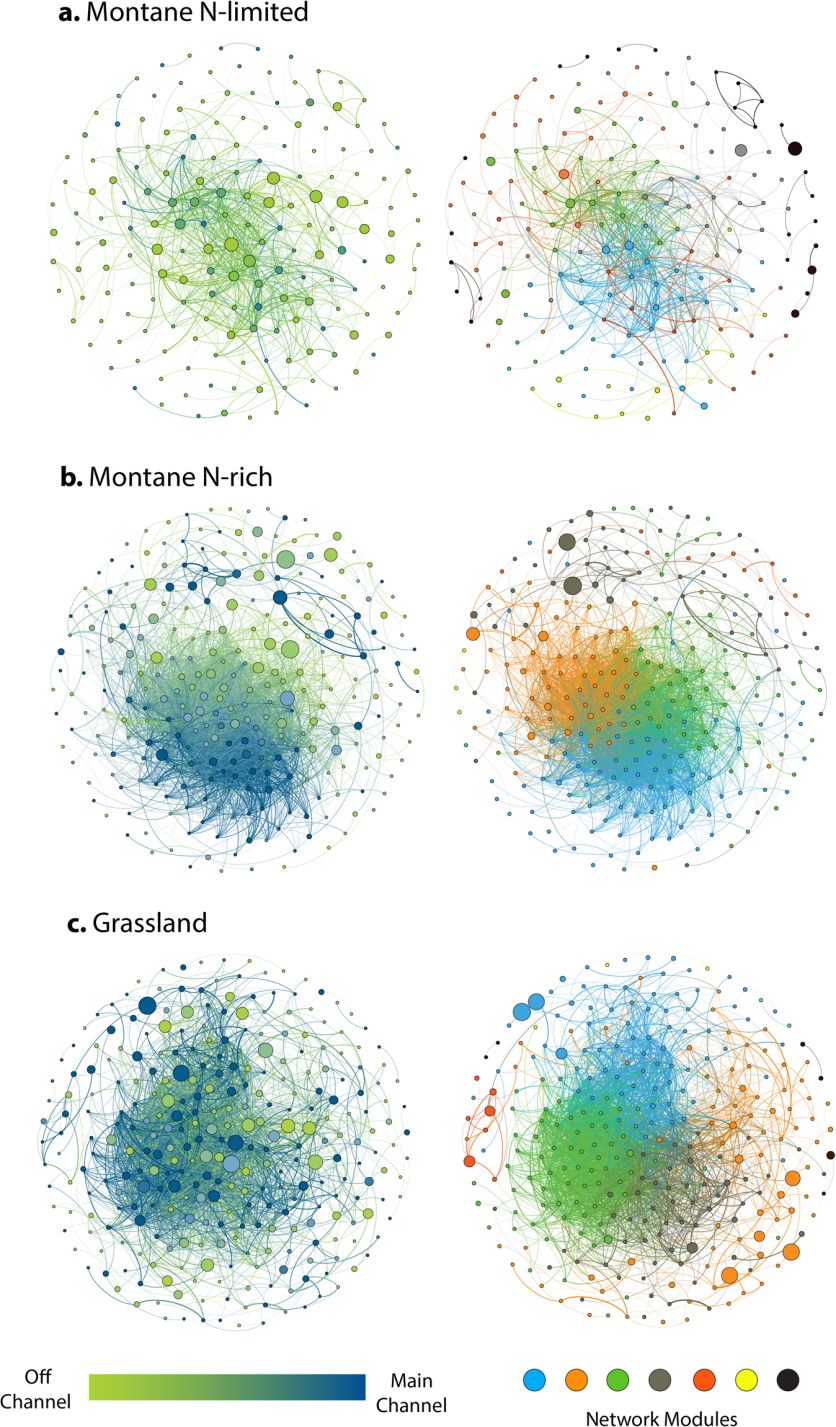
Co-occurrence microbial networks. Computed co-occurrence networks for river-floodplain systems in the N-limited (**a**), N-rich (**b**), and grassland (**c**) sub-biomes. Nodes in networks on the left are color coded to reflect relative abundance in main- and off-channel zones, while node sizes are proportional to values of betwenness centrality. Nodes in networks on the right are color coded to reflect the different bacterial modules to which they belong, with large (i.e., higher number of nodes) and small (representing less than 2.5% of the network nodes) modules in blue and black, respectively. Node size is proportional to OTU relative abundance. An interaction (edge) reflects a significant Pearson correlation (-0.6 > R > 0.6 and p-value ≤ 0.01) with edge thickness proportional to correlation coefficients.

As guided by results from unconstrained ordination, we computed a total of three co-occurrence bacterial networks congruent with recognition of the sub-biomes characterized by flow regimes and biogeochemical conditions. Generating independent networks for the combined rivers within the grassland biome and for the two biogeochemical settings in the montane biome allowed sufficient sample size and reliability for network analysis (Table 2). Total number of nodes (*i*.*e*., OTUs) in the co-occurrence network of N-limited floodplains was lower than for bacterial networks of grassland or montane N-rich floodplains (Table 2; Fig. 5a–c). Even greater differences were found for the total number of interactions (*i*.*e*., edges) among nodes, which tripled in grassland and N-rich communities compared to in N-limited bacterial communities (Table 2; Fig. 5). In grassland and N-rich montane networks, nearly every (98 & 99%, respectively) bacterial interaction was positive, whereas more than a quarter of the correlations found in the N-limited network were negative (Table 2). Moreover, at the zone level (*i*.*e*., main-channel vs. off-channel) most of the interactions in grassland and N-rich montane floodplains involved taxa whose relative abundance co-varied positively across floodplain zones (Fig. 5b,c). In contrast, approximately half the bacterial interactions in N-limited floodplains involved pairs of OTUs with opposite zonal patterns in relative abundance (Fig. 5a).

**Table 2:** Biofilm network parameters.

Semi-biome	Nodes (no.)	Edges (no.)	Node Degree	Closeness Centrality	Betwenness Centrality	Keystone Taxa (no.)	Positive edges (%)	Negative Edges (%)	Modularity
Montane (N-limited)	178	781	4 (2, 13)	0.32 (0.27, 0.38)	0.0008 (0.000, 0.019)	23	72 (335, 229)	28 (91, 124)	0.374
Montane (N-rich)	273	3669	12 (3, 45)	0.36 (0.29, 0.41)	0.003 (0.000, 0.011)	32	99 (2773, 597)	1 (6, 33)	0.212
Grassland	299	3438	15 (4, 39)	0.36 (0.31, 0.42)	0.004 (0.000, 0.009)	44	98 (2773, 597)	2 (51, 17)	0.286

Central tendencies and variation of the computed network parameters to quantify network complexity in the grassland biome (n = 27), montane N-rich sub-biome (n = 37), and montane N-limited sub-biome (n = 54). Values for average degree, closeness centrality, and betwenness centrality are expressed as median (25^th^, 75^th^ percentile). For the percentage of positive and negative interactions (edges), the amount of edges connecting OTUs associated with the same floodplain zone and the amount of edges connecting OTUs associated with different zones (main- and off-channel) are also indicated within parenthesis.

Network connectivity–Four parameters were assessed to estimate node connectivity with the wider network including node degree, closeness centrality (CC), betweenness centrality (BC), and keystone species designation. Node degree represents the average amount of edges per node in each network, a measure much lower and less variable in N-limited biofilms than in the other networks (Table 2). Similarly, CC (measured as the inverse of total distance to other nodes) was lowest in N-limited communities indicating a larger fraction of OTUs poorly connected to the larger network (Fig. 5a–c). Median betweenness centrality (BC; fraction of times a node is found on the shortest path between two nodes) was also lowest in the N-limited network (Table 2), but a few nodes in this sub-biome showed a disproportionate influence on the interactions between other taxa (note high 75^th^ percentile BC values in Table 2; Fig. 5a). The proportional abundance of keystone taxa (*i*.*e*., nodes exceeding the 75^th^ percentile of node degree, CC, and BC) was similar among networks (12–15% of all nodes; Table 2) without propensity to occur within a specific floodplain zone, and thus indicating that keystone taxa are equally likely to be found in main channel and floodplain habitats (Fig. 5a–c; Table S5). The relative abundance of keystone taxa in each habitat (as fraction of total reads) was positively correlated with sample taxon richness in grassland (R = 0.83; *p*-value < 0.001) and N-rich (R = 0.87; *p*-value < 0.001) communities, but not in N-limited floodplains (Fig. S4).

Network modularity–The degree to which a network is composed of sets of nodes highly connected to each other but poorly connected to other sets describes network modularity. A total of 7 modules were identified in each network encompassing more than 95% of the total number of nodes. Greater modularity occurred in N-limited biofilms compared to N-rich and grassland communities (Table 2). High network modularity in N-limited conditions is illustrated by fewer connections among taxa belonging to different modules (Fig. 5d), contrasting with the numerous linkages across modules in N-rich and grassland assemblages (Fig. 5e,f). With few exceptions, more abundant community members were included in one or more of the larger network modules (*i*.*e*., those that include a greater number of nodes). However, in grassland and N-rich communities, abundant taxa were rather poorly connected (low node degree and BC) and located at the edge of their respective modules (Fig. 5d–f), while most abundant taxa in N-limited communities were generally more strongly connected within each module (Fig. 5d). For the N-limited floodplains, two abundant Cyanobacteria families–unnamed families but one mostly representing *Leptolyngbya* and *Microcoleus*, and the other *Calothrix* and *Nostoc* genera–were the most well-connected taxa in the second largest module (orange in Fig. 5d). One of these cyanobacteria families was also identified as a keystone taxon (Table S5) and its abundance was strongly correlated (R = 0.73; P-value < 0.001) with the relative abundance of heterocystous cyanobacteria across all biofilm samples. Together, these results suggest a central role of N-fixing cyanobacteria in microbial networks of river-floodplain systems under N limiting conditions (Figs 5d and S5).

## Discussion

As human pressures on riverine landscapes continue to increase, understanding the consequences of floodplain simplification for freshwater biodiversity is of high priority^42^. Hypothesized losses of species diversity due to degradation of floodplain habitat diversity are supported by extensive evidence from a variety of taxa^43–45^. But the validity of this hypothesis when applied to microbial communities is far less resolved and was the main rationale for this study. We gathered little evidence for niche differentiation at the habitat scale that leads to unique microbial communities within floodplains. Biophysically complex floodplains supported alpha- and beta-diversity similar to that in grassland floodplains with limited habitat heterogeneity. The lack of significant differentiation of biofilm communities among the array of aquatic habitats surveyed contrasts with the occurrence of distinct biogeochemical mixtures of C, P, and N as central determinants for biofilm community differentiation within and across riverine floodplains. Hence, our results do not generally support the idea that increased habitat diversity and environmental variation in riverine floodplains lead to more diverse biofilm communities. Instead microbial composition was organized at the scale of sub-biomes and among the floodplains within them, reflecting biogeochemical and geographic character of entire river-floodplain systems and across large, regional scales that contain them.

Partitioning biofilm communities into universal groups may be impossible to determine objectively, but practical divisions can prove informative. Identifiable communities based on assemblage structure and interactions across landscapes have been used, for instance, to describe how catchment properties relate to changes in bacterial communities in a context of microbial biogeography^13,46^. Our results suggest a similar approach may be taken to assess regional influences on microbial communities of floodplain landscapes. Biofilm community composition in riverine floodplains is driven by regional gradients in geochemical conditions more than endogenous environmental gradients provided by floodplain habitat diversity. Of central importance to that geochemical template is the relative and absolute availability of N and P, fundamental resource pools that may act as master variables for species sorting. Regional flow regimes and gradients of nitrogen limitation exerted strong influences on species sorting and interactions of biofilm assemblages, emphasizing spatial niche differentiation at large scales.

### River flow, floodplain complexity, and biofilm diversity

River impoundments alter natural flow regimes and reduce lateral hydrologic connectivity while decreasing the abundance and diversity of floodplain habitats such as backwaters, springbrooks, and ponds^18,42,47^. By selecting floodplains in relatively free-flowing rivers and others located downstream of dams, we intended to capture a gradient of habitat complexity and environmental heterogeneity with subsequent implications for biofilm communities. Our simple analysis of flow alteration showed that river dams reduced flow predictability, a major organizing factor for lotic communities^30^. As expected, declining flow predictability was strongly correlated to reduction in the diversity of aquatic habitats found in each floodplain, and to biogeochemical variation among habitats where epilithic biofilms develop. Together these patterns are indicative of river and floodplain simplification associated with river dams^18^. Results support our initial premise that more natural flow regimes translate into greater floodplain complexity and stronger environmental gradients, but increased environmental heterogeneity alone did not strongly translate to greater bacterial co-existence and enhanced microbial biodiversity.

Biofilm diversity (as average α-diversity) was highest in more regulated rivers (*e*.*g*., Big Horn River) with lower habitat diversity and biogeochemical variation, and the degree of community differentiation (β-diversity) was unrelated to the range of abiotic conditions observed within each floodplain. These results suggest similar degrees of community differentiation among bacterial taxa with varying environmental tolerances and among riverine floodplains of contrasting habitat diversity. Hence, processes of niche partitioning promoting species co-existence for communities of macro-organisms of floodplain landscapes^20–25^ may not equally apply to biofilm communities. One may argue that niche partitioning in biofilm assemblages may be obscured by scale effects on ecological phenomena. Previous work at small scales (10^−1^ m) has demonstrated how increased flow and substrate heterogeneity can enhance both β-diversity^37^ and functional diversity^48,49^. Heterogeneous habitat across floodplain landscapes manifest at spatial scales in the order of 10^2^–10^3^ m, likely larger than the spatial dimension evoking microbial responses^50^. Although our experimental designed allowed us to compare habitat and bacterial diversity across biome, floodplain, zone, and habitat scales, it is possible that our finest level of resolution was too coarse to discern niche differentiation in microbial assemblages^36,51^. However, the argument of a large-scale decoupling between environmental variation and microbial responses contrasts with regional abiotic controls on aquatic biofilms found in this and previous studies on similar ecosystems and scales^13,52,53^.

### Environmental controls on biofilm assemblages of riverine floodplains

Differences in community structure were clearly greater among floodplains than at any other spatial scale. Similar patterns were found for the strongest predictors of biofilm community structure (*i*.*e*., DOC and SRP) that differed more strongly across floodplains than within them. Riverine floodplains represented distinct spatial units distributed across regional environmental gradients, leading to ubiquitous occurrence of bacterial taxa with similar environmental tolerances^52^ despite quantifiable heterogeneity and complexity at the habitat scale. Recognizing it as the fundamental energy source to heterotrophic microbes, a great number of studies have demonstrated how quality and quantity of DOC can significantly affect biofilm community composition^54^, as well as microbial contribution to ecosystem processes^55,56^. Biogeochemical distinctions were also tied to SRP and N availability manifested as regional gradients of N to P ratios which led to changes in the abundance of cyanobacteria and eukaryotes, and properties of microbial co-occurrence networks. Our results illustrated striking dissimilarity in community structure between montane sub-biomes of contrasting N and P availability. Regional variation in P concentrations can be attributed to the existence of P-rich geologic formations in southwest Montana^57^, leading to riverine systems perennially enriched in SRP, promoting N-limitation of biological processes when N demand increases^58^. This was the case for the Boulder, Clark Fork, and Madison Rivers, and to a lesser extent, the Bitterroot River. Under conditions of high water temperature, high P availability and low N concentrations, heterocystous cyanobacteria with the ability to fix atmospheric-N heavily influence the structure and function of lotic ecosystems^59,60^. Significant increases in N-fixing cyanobacteria, distinct community structure, and unique network topology in SRP enriched (*i*.*e*., low-N) systems suggest a critical role for N-limitation in structuring composition, function, and negative species interactions of biofilm communities.

### Nitrogen limitation and microbial networks in river floodplains

Microbial communities in floodplain landscapes are proposed to function as meta-communities^61,62^, in which local extinctions due to direct competition can be rapidly overcome by recolonization from nearby habitats^63^. Hence, niche differentiation in the form of environmental control on community assembly should have strong influences on local community structure^64^. Co-occurrence of microbial taxa across floodplain habitats is likely to capture those unique responses of microbial taxa to local environmental conditions. Positive co-occurrence is thought to reflect interactions between ecologically similar taxa^65^ that require similar environmental settings and resources^66^, and likely perform similar or complementary functions. By contrast, negative interactions are indicative of direct competition for resources among microbial taxa, or the result of resource partitioning^67^. Agler, *et al*.^68^ provided compelling results supporting these interpretations by showing how positive co-occurrence accounted for 86% of the interactions among microbial taxa of the same kingdom (fungi or bacteria), while only 23% of cross-kingdom interactions were positive. A dominance of positive interactions in microbial networks has been commonly observed in benthic biofilms^53,69^, plant microbiomes^68^, soil communities^70,71^, or marine bacterioplankton^67^. In our study, positive interactions dominated each of the three computed networks, but more notably when N was not limiting biological activity. We relate the increased proportion of negative interactions in N-limited floodplains to increased resource competition along extreme gradients of N-limiting conditions as described by N:P ratios that differed by orders of magnitude within and among floodplains. Nitrogen fixation is highly sensitive to changes in abiotic controls with rapid, nonlinear responses when thermal or nutrient thresholds are excedeed^59,72,73^, providing a significant competitive advantage to N-fixing bacteria when favorable conditions (high temperature, P, and low N) are met^52^. Further effects of increased resource competition on N-limited biofilms may be the lack of positive relationships between keystone taxa abundance and bacterial richness observed in other N-rich rivers of grassland and montane biomes. Positive co-occurrence exists among keystone species and other taxa directly or indirectly affected by them, likely taxa requiring similar environmental settings or performing complementary functions^65,68^, resulting in overall increases in taxa richness^74^. But in riverine floodplains affected by regional constraints of low N availability and concomitant P enrichment, patterns of bacterial richness were more likely influenced by local increases in the relative abundance of N-fixing cyanobacteria that possessed a clear competitive advantage in the use of the limiting nutrient resources. This interpretation is further supported by increased network modularity in N-limited floodplains and the central role of cyanobacteria in the largest modules (sub-networks). Two of the most abundant cyanobacteria families (representing 10% of total reads) were found in the second largest module and included many genera with the ability to fix N (*e*.*g*., *Nostoc*). Networks with more modular structures result from the emergence of distinct network units (*i*.*e*., modules) coping with resource constraints and unique niche opportunities^75,76^, which can lead to greater abundance of negative relationships in the network^77^ as we observed in N-limited communities. Our results indicate that effects of niche partitioning associated with N-limiting conditions can lead to increased modularity of epilithic biofilm communities^78^, in which N-fixing cyanobacteria may play a central role in determining assembly and network structure for biofilm communities when regional constraints on N and P availability increase resource competition among and within floodplain habitats of N-poor sub-biomes.

## Methods

### Field sampling

A nested sampling design was employed to capture spatial organization of river-floodplain systems at different scales. First, a *ca*. 10 km of floodplain in each river system was selected and equally divided as upper, middle, and lower sections that were sequentially surveyed over a three-day period. Within each section, lateral affiliation was characterized by ‘main-channel’ and ‘off-channel’ zones. Five aquatic habitats within each zone were sampled when present: *riffles*, *runs*, *pools*, *channel confluences*, and *shoreline* within the boundaries of main-channel zone; and *backwaters*, *side channels*, *parafluvial springbrooks*, *orthofluvial springbrooks*, and *ponds* in the off-channel zone with varying distances to the river’s main channel. Detailed definitions of each habitat type can be found in Peipoch, *et al*.^52^. A total of 265 aquatic habitats were surveyed for biogeochemical characterization (details described below) during baseflow conditions (Fig. 1b). Epilithic biofilms were present in approximately half of the surveyed habitats (n = 126), from which composite samples for 16S- rRNA sequencing were collected.

Four out of ten rivers [*Bitterroot*, *Clark Fork*, *Boulder*, and *Madison]* were sampled during July-August of 2013 and six [*Swan*, *Big Hole*, *Bighorn*, *Tongue*, *Milk*, and *Missouri*] were collected during June-August of 2014. Water samples for inorganic and total dissolved nutrients were collected in triplicate at each habitat and immediately filtered (0.7-μm pore size, glass fiber filter, Whatman International, Kent, UK), kept on ice during transport, and frozen until further analysis in the laboratory. Samples for the analysis of chlorophyll and organic matter (i.e., ash-free dry mass, AFDM) were collected as composite scrapes from three to five different cobbles of known area within each habitat and stored on ice under dark conditions until further processing in the laboratory. Biofilms from 15–20 different locations within sampled habitats were carefully scrapped from rock surfaces using sterile disposable spatulas to generate a composite sample that was stored in 2-mL Safe-Lock microcentrifuge tubes (Eppendorf AG, Germany). Samples were kept on ice during transport centrifuged at 13000 rpm for 5 minutes to remove excess water, and frozen at −20 °C until DNA extraction processing.

### Analytical methods

Dissolved organic carbon (DOC) concentrations were determined using an Aurora 1030 W TOC Analyzer (Oceanographic Int., College Station, Texas, USA) and chemical oxidation methods^79^. The concentrations of ammonium-nitrogen (N-NH_4_), nitrate-nitrogen (N-NO_3_), and Soluble Reactive Phosphorous (SRP) were determined by micro-segmented flow analysis using an Astoria 2 Analyzer (Astoria-Pacific Inc., Clackamas, Oregon, USA) following standard procedures^80^. Determination of total nitrogen (TN) and total phosphorous (TP) was conducted by performing an alkaline persulfate digestion to each sample followed by analysis of N-NO_3_ and SRP using the same instrumentation and methods detailed above^81^. Biofilm samples for BOM as AFDM were dried at 100 °C for 24 h, weighed as dry mass, subsampled if necessary, combusted at 550 °C for 6 h and weighed as ash. Benthic chlorophyll-*a* was determined by macerating samples in 90% acetone for 24 h and measuring extract absorbance in a V-550 UV/VIS spectrophotometer (Jasco Inc., Easton, Maryland, USA) before and after acidification to allow proper corrections for phaeopigments^82,83^.

### DNA extraction and sequencing

Deoxyribonucleic acid (DNA) was extracted from biofilms using the PowerBiofilm® DNA Isolation Kit (MO BIO Lab. Inc., Carlsbad, California, USA) following manufacturer instructions. The V4 region of the16S rRNA gene was amplified in triplicate using primers F515/R806 tagged with 12-base Golay codes^84^. Samples from rivers surveyed in 2013 and 2014 were sequenced using the Illumina MiSeq platform at Argonne National Laboratory and Genomics Core Laboratory of the University of Montana, respectively. Approximately 250 base-pair reads were generated from each direction, resulting in nearly the entire targeted region having double coverage. Reads were assembled, Organizational Taxonomic Units (OTUs) were generated based on 97% sequence similarity, and chimeras were removed using USEARCH v7.0. For data analysis we used both MOTHUR [https://aem.asm.org/content/75/23/7537.short] and QIIME2 [https://peerj.com/preprints/27295/]. For each application we did 3 analyses: for the samples in 2013, for the samples in 2014, and for a mix of both years. We did this because the amount of information available on the two different sampling events was quite different, with orders of magnitude more data being available for the sampling of 2014. Also the protocol followed was slightly different – the data were available demultiplexed from 2014 whereas 2013 required demultiplexing. The analysis with MOTHUR provided more consistent results than QIIME2 in that the relative proportions of the mixed analysis were in line with the observations from the analysis of the individual years. In addition, we also validated that the relative proportions estimated by MOTHUR and QIIME2 analysis were consistent for each of the years when taken in isolation. Overall, we followed a slightly adapted version of the MiSEQ SOP available at https://mothur.org/wiki/MiS. Prior to data analysis of the bacterial community, sequences identified as chloroplasts were removed from the dataset (but see further analysis of chloroplasts sequences below). All OTUs present in less than a quarter of the total samples and/or with less than 20 sequences were also removed. Each sample was then rarefied to contain 10,000 sequences.

Then, and because MOTHUR does not provide taxonomic assignments for chloroplast sequences, we used QIIME to compare the relative abundances of different photosynthetic microorganisms. Following the identification of different algal groups with QIIME, we conducted downstream analyses of streptophyte and stramenopile algal OTUs, respectively. For streptophyte OTUs, we used local nucleotide BLAST (blastn) searches to distinguish plant versus algal OTUs; approximately 82% of streptophyte sequence reads (268,623/326,888) were derived from plants and were not included in further analyses. We also used blastn searches against the NCBI GenBank nr database to assign stramenopile OTUs to different classes (*e*.*g*., Bacillariophyceae (diatoms), Phaeophyceae (brown algae) etc.).

### Data analyses

Variables were log-transformed to meet normality and homodestacity requirements before any of the analysis performed. Variance component analysis for each environmental variable was performed using restricted maximum likelihood estimations with the ‘remlVCA’ of the *VCA* package in order to identify the specific spatial scale at which each biotic and abiotic variable was varying most. Principal Component Analysis (PCA) was performed using the ‘nipals’ function from the *ade4* package to characterize environmental variation within and among riverine floodplains. Environmental heterogeneity of each floodplain was calculated as the mean and coefficient of variation (CV) of distance from each habitat site to the floodplain group centroid in the PCA ordination using the ‘betadisper’ function of the *vegan* package. We explored biofilm community similarity at the Family level using Non-metric Multidimensional Scaling (NMDS) based on Bray-Curtis dissimilarity calculated by the ‘metaMDS’ function from the *vegan* package. Statistical differences in community similarity among groups were tested using the ‘anosim’ function, and environmental variables were fitted into the NMDS plot to facilitate interpretation and guide further analyses without incorporating environmental constraints using the ‘envfit’ function. Beta diversity (dissimilarity between communities of different sites) was calculated for each floodplain as the sum of its two components or phenomena, *nestedness* of species assemblages (*i*.*e*., species in specific habitats being subsets of communities from richer habitats) and *spatial turnover* (species replacement among habitats), following procedures and methods detailed in the ‘beta.pair.abund’ function of the *betapart* package^85^. Contributions of specific taxa to floodplain’s beta diversity were estimated using the ‘beta.div’ function of the *vegan* package.

We used partial Redundancy Analysis (pRDA) to assess environmental controls on biofilm community composition while accounting for spatial effects on community similarity. We first generated distance-based Moran’s Eigenvector Maps (db-MEMs) following procedures described by Dray, *et al*.^86^. In brief, db-MEMs characterize spatial variation among sites by generating eigenvector-based variables (similar to PCA eigenvalues) that can be later used as predictors for community patterns. Geographic distances used as input for db-MEMs were calculated using Euclidean distances and UTM coordinates. Once spatial variables were obtained, we performed an RDA to exclude those that were not significantly explaining patterns of community similarity using the ‘rda’ and ‘ordiR2step’ functions from *vegan* package. Then, a pRDA was done using the ‘varpart’ function of vegan to estimate individual and shared explanatory power of environmental and geographic distances on biofilm community structure. Adjusted R^2^ values were used to evaluate the specific contribution of environmental and spatial predictors of biofilm assemblages. Relationships among environmental heterogeneity, microbial diversity, and network properties at multiple scales were explored using regression analysis. All statistical analyses were performed using the R software version 3.5.0^87^.

To ensure network analysis was performed out of nonrandom co-occurrence interactions we first calculated a C-score index^88^ and compared it to a null model with fixed OTU/site frequencies using the ‘ecospat.Cscore’ function from *ecospat* package^89^. Then, co-occurrence interactions among microbial taxa were examined at a 90% identity threshold corresponding roughly to a taxonomic level of Family^90^ for the sake of network visualization^28,77^. Valid co-occurrence associations were considered when pairwise correlations were equal or in excess of 0.6 or −0.6, and statistically significant (*p-*value < 0.01). All the necessary steps for network inference were performed in the R environment (http://www.r-project.org) using *igraph* package^91^. Networks were then visualized and examined using the gephi platform^92^. To assess network structure we calculated average node degree and network’s modularity following Newman^93^ procedures. Node degree corresponds to the average number of edges per node and modularity quantifies the abundance of modules (set of nodes highly connected to each other while poorly connected to nodes of other groups) in the network. We identified keystone taxa (aka hubs) in each network when their values of node degree, closeness centrality, and betweenness centrality exceeded the 75^th^ percentile of each of those parameters. Closeness Centrality is inversely proportional to the sum of distances to each other node of the network and Betweenness Centrality is equal to the number of times a node is in between the shortest path between any two nodes in the network. Together, node degree, CC, and BC values are indicative of the propensity of an OTU to act as keystone taxa^94^.

Sequencing data derived from this study are available as BioProject PRJNA551494at in https://www.ncbi.nlm.nih.gov/.

## Supplementary information

Supplementary Information
