# Supplementary material for: Niche partitioning of microbial communities in riverine floodplains
Source: Sci Rep. 2019 Nov 8;9:16384. doi: 10.1038/s41598-019-52865-4 (PMC6841707; doi:10.1038/s41598-019-52865-4)
Supplement: Supplementary file 1 — Supplementary Information [file 41598_2019_52865_MOESM1_ESM.docx]

Supplementary Information

**Niche partitioning of microbial communities in riverine floodplains**

Marc Peipoch^1*^, Scott Miller^2^, Tiago Antao^2^, and H. Maurice Valett^2^

^1^Stroud Water Research Center, Avondale, PA

^2^Division of Biological Sciences, University of Montana, Missoula, MT

^*^mpeipoch@stroudcenter.org

**Figure S1 | Discharge records.** Long-term records (1970-2010) of daily discharge (m^3^ s^-1^) in the selected river systems. Data obtained from USGS gauging station located nearby our study sites. Panels (a-e) correspond to the riverine floodplains without the influence of upstream river dams: Boulder (a), Clark Fork (b), Big Hole (c), Swan (d), and Bitterroot (e) Rivers. Panels (f-j) correspond to riverine floodplains located downstream of impoundments: Milk (f), Tongue (g), Madison (h), Bighorn (i), and Missouri (j) Rivers.

**
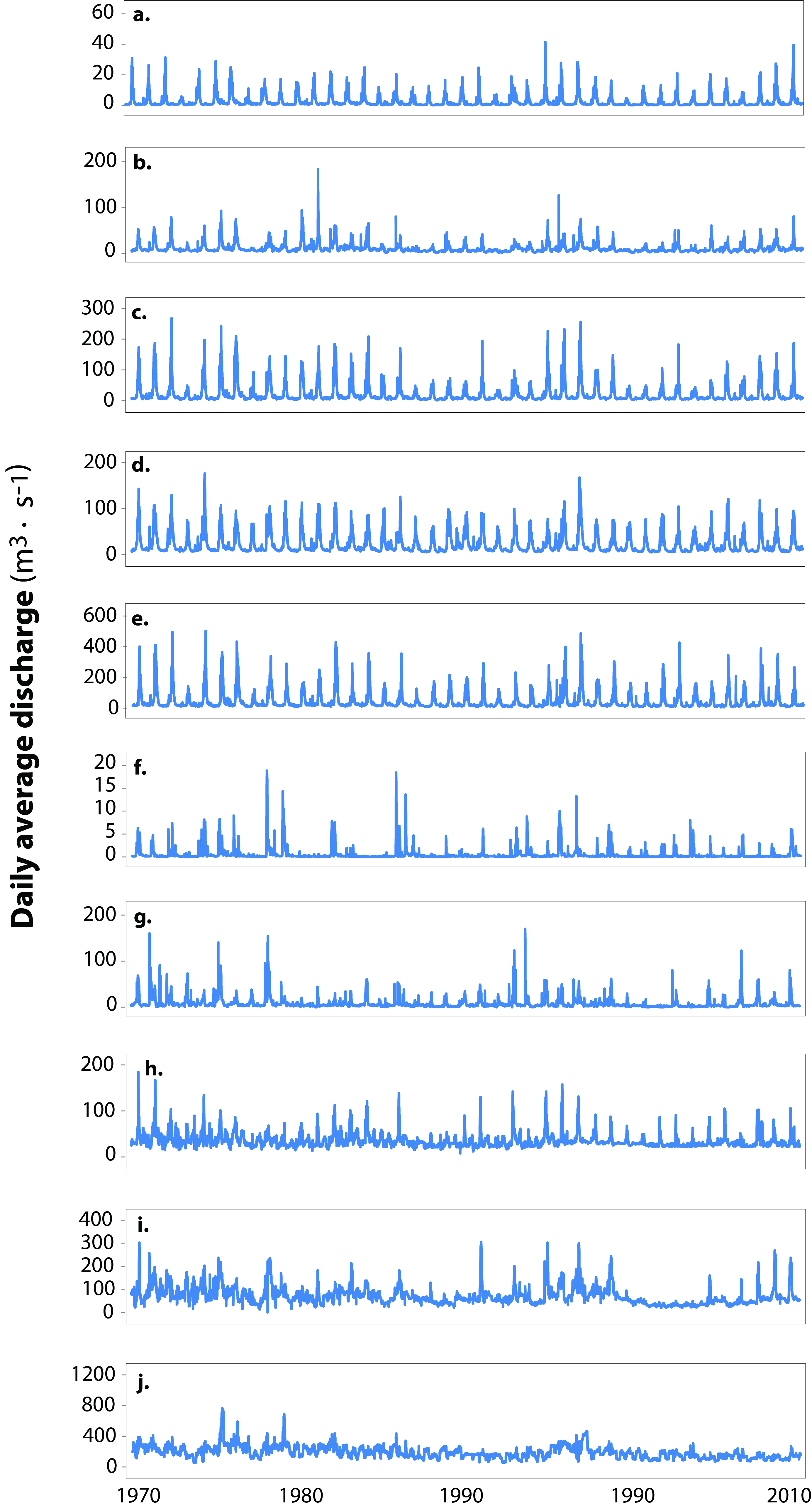
**

| **Table S1 \| Multi-scale environmental variation.** Mean values and standard error of the mean (SEM) of chemical and biological conditions at biome, floodplain, zone, and habitat scales. Superscripts indicate significant differences in chemical and biological conditions calculated by Tukey HSD tests (p-value <0.5). | | | | | | | | | | | | | | | | | |
| --- | --- | --- | --- | --- | --- | --- | --- | --- | --- | --- | --- | --- | --- | --- | --- | --- | --- |
|  | | **N** | **DOC** (mg/L) | **N-NH_4_** (µg/L) | **N-NO_3_** (µg/L) | **DON**  (µg/L) | | **TN**  (µg/L) | | **SRP**  (µg/L) | | **TP**  (µg/L) | **molar NP** (unitless) | **Chl-a** (mg/m2) | | **BOM** (g/m2) | |
| **Biome** | |  |  |  |  | |  | |  |  |  | |  | |  | |  |
| Grassland | | 98 | 5.1 ± 0.3 | 20.2 ± 5.3 | 54.1 ± 10.2 | 318 ± 32.5 | | 388 ± 33.3 | | 13.7 ± 1.9 | | 20.7 ± 3.3 | 31 ± 5.9 ^a^ | 55.3 ± 46.2 | | 30.7 ± 4.7 ^a^ | |
| Montane | | 163 | 3.9 ± 0.3 | 12.2 ± 2.6 | 20.5 ± 6 | 235 ± 16 | | 266 ± 17.4 | | 36.7 ± 1.9 | | 29.6 ± 2.5 | 5 ± 1 ^b^ | 81.4 ± 13.4 | | 219 ± 140.9 ^b^ | |
|  | |  |  |  |  |  | |  | |  | |  |  |  | |  | |
| **Floodplain** | |  |  |  |  | |  | |  |  |  | |  | |  | |  |
| Boulder | | 29 | 3.1 ± 0.2 ^cd^ | 9.7 ± 2 ^bc^ | 7.3 ± 1.5 ^c^ | 158 ± 10.9 ^c^ | | 175 ± 10.1 ^c^ | | 54.5 ± 2.1 ^ab^ | | 23.6 ± 3.5 ^b^ | 0.7 ± 0.1 ^e^ | 61.4 ± 31.1 ^b^ | | 97.1 ± 42.2 ^bc^ | |
| Clark fork | | 28 | 2.9 ± 0.3 ^d^ | 22.9 ± 10.7 ^a^ | 6.6 ± 3.2 ^c^ | 294 ± 41.4 ^b^ | | 323 ± 41.7 ^b^ | | 42 ± 3.3 ^b^ | | 39.6 ± 7.3 ^b^ | 2.1 ± 1.1 ^de^ | 196 ± 52.3 ^a^ | | 131 ± 21 ^b^ | |
| Big Hole | | 24 | 12 ± 0.7 ^a^ | 6.6 ± 2.5 ^bc^ | 58.3 ± 38 ^b^ | 546 ± 30.1 ^a^ | | 608 ± 36.3 ^a^ | | 42.2 ± 1.9 ^b^ | | 71 ± 4.6 ^a^ | 3.6 ± 2.2 ^cd^ | 10.3 ± 5.1 ^c^ | | 39 ± 12.2 ^c^ | |
| Swan | | 28 | 1.3 ± 0.1 ^e^ | 3.2 ± 1.3 ^d^ | 30.8 ± 11.1 ^b^ | 55.2 ± 5.7 ^d^ | | 78.6 ± 6.3 ^d^ | | 4.9 ± 0.4 ^e^ | | 3.1 ± 0.6 ^d^ | 18.2 ± 4.8 ^b^ | 57.3 ± 26.5 ^b^ | | 70.9 ± 33 ^c^ | |
| Bitterroot | | 30 | 2.7 ± 0.4 ^d^ | 6 ± 1.1 ^c^ | 18.5 ± 3.1 ^b^ | 193 ± 26.9 ^bc^ | | 219 ± 28 ^bc^ | | 15.2 ± 1.8 ^c^ | | 21.3 ± 5.6 ^b^ | 4.2 ± 0.6 ^c^ | 50 ± 13.9 ^b^ | | 46.2 ± 14.3 ^c^ | |
| Milk | | 25 | 6.9 ± 0.4 ^b^ | 33.5 ± 16.1^a^ | 4.9 ± 1 ^d^ | 306 ± 39 ^b^ | | 346 ± 38.3 ^b^ | | 23.8 ± 6.7 ^c^ | | 31.4 ± 9.5 ^b^ | 4.1 ± 1.6 ^c^ | 2.2 ± 2 ^d^ | | 20.4 ± 6.2 ^a^ | |
| Tongue | | 27 | 4.9 ± 0.3 ^c^ | 13.4 ± 4 ^b^ | 5.8 ± 1.9 ^cd^ | 251 ± 50.1 ^b^ | | 267 ± 48.8 ^b^ | | 9.7 ± 0.8 ^d^ | | 14.6 ± 3.8 ^c^ | 4.5 ± 1 ^c^ | 2.5 ± 0.9 ^d^ | | 20.6 ± 7.2 ^d^ | |
| Madison | | 24 | 2.5 ± 0.2 ^d^ | 26.6 ± 11.1 ^a^ | 2.6 ± 0.5 ^d^ | 174 ± 21.9 ^c^ | | 202 ± 31.2 ^c^ | | 67.2 ± 1.6 ^a^ | | 19 ± 1.5 ^bc^ | 0.9 ± 0.3 ^e^ | 118 ± 37.4 ^ab^ | | 1023 ± 931 ^a^ | |
| Big Horn | | 21 | 3.8 ± 0.3 ^c^ | 22.2 ± 14.4 ^ab^ | 234 ± 17.3 ^a^ | 298 ± 56.3 ^b^ | | 538 ± 50.5 ^b^ | | 6.5 ± 1.6 ^e^ | | 17.6 ± 4.7 ^c^ | 130 ± 12.6 ^a^ | 15.7 ± 6.1 ^c^ | | 29 ± 6.6 ^cd^ | |
| Missouri | | 25 | 4.8 ± 0.8 ^c^ | 12.6 ± 2.3 ^b^ | 4 ± 0.8 ^d^ | 369 ± 78.3 ^b^ | | 386 ± 78.7 ^b^ | | 14.2 ± 1.9 ^c^ | | 16.1 ± 3.3 ^c^ | 2.9 ± 0.6 ^d^ | 18.8 ± 3.5 ^c^ | | 52.8 ± 13.7 ^c^ | |
|  | |  |  |  |  |  | |  | |  | |  |  |  | |  | |
| **Zone** | |  |  |  |  | |  | |  |  |  | |  | |  | |  |
| Main-channel | | 145 | 4 ± 0.3 | 9 ± 1.9 ^a^ | 28.9 ± 6.3 | 233 ± 20.1 | | 267 ± 20.5 | | 24.1 ± 1.8 | | 18.6 ± 1.5 ^a^ | 16.5 ± 3.6 | 29.1 ± 8.8 ^a^ | | 24.4 ± 4.4 ^a^ | |
| Off-channel | | 116 | 4.9 ± 0.4 | 23 ± 5.2 ^b^ | 38.4 ± 9.5 | 296 ± 22.7 | | 349 ± 25.5 | | 33 ± 2.6 | | 36.7 ± 3.9 ^b^ | 12.7 ± 3.2 | 113 ± 33.4 ^b^ | | 281 ± 181.7 ^b^ | |
|  | |  |  |  |  |  | |  | |  | |  |  |  | |  | |
| **Habitat** | |  |  |  |  | |  | |  |  |  | |  | |  | |  |
| Riffle | | 25 | 4 ± 0.7 ^bc^ | 10.7 ± 3.6 ^b^ | 33.3 ± 18.4 | 201 ± 36.5 ^cd^ | | 245 ± 37.7 ^cd^ | | 26.9 ± 4.3 ^b^ | | 20.3 ± 3.9 ^b^ | 15.5 ± 8.2 ^a^ | 25.5 ± 8.5 ^b^ | | 30.6 ± 12.8 ^b^ | |
| Run | | 47 | 4.8 ± 0.5 ^b^ | 10.1 ± 1.7 ^b^ | 20.4 ± 8.7 | 244 ± 24.4 ^bc^ | | 264 ± 26 ^bc^ | | 20.3 ± 2.7 ^b^ | | 19 ± 2.7 ^b^ | 13.9 ± 5.9 ^a^ | 40.1 ± 24.3 ^ab^ | | 20.8 ± 7.2 ^b^ | |
| Pool | | 27 | 3.4 ± 0.4 ^c^ | 3.7 ± 0.6 ^c^ | 38.5 ± 17.8 | 215 ± 28 ^cd^ | | 261 ± 32.2 ^cd^ | | 21.6 ± 4 ^b^ | | 16.1 ± 2.8 ^bc^ | 20.5 ± 10 ^a^ |  | |  | |
| Confluence | | 21 | 4 ± 0.8 ^b^ | 5.2 ± 1.5 ^c^ | 35.9 ± 17.8 | 333 ± 104.6 ^a^ | | 361 ± 103.6 ^a^ | | 30.5 ± 5.4 ^a^ | | 22.2 ± 5.5 ^b^ | 22.2 ± 10.4 ^a^ | 25.6 ± 9.6 ^b^ | | 30 ± 9.8 ^b^ | |
| Shoreline | | 26 | 3.3 ± 0.4 ^c^ | 13.6 ± 9.4 ^b^ | 23.3 ± 11.8 | 183 ± 27.6 ^d^ | | 223 ± 30.4 ^d^ | | 24.8 ± 4.5 ^b^ | | 15.8 ± 2.6 ^c^ | 12.6 ± 7 ^ab^ | 18.6 ± 4.6 ^b^ | | 22.2 ± 6 ^b^ | |
| Backwater | | 29 | 4.3 ± 0.5 ^b^ | 21.6 ± 10.6 ^ab^ | 45.6 ± 16 | 301 ± 41.9 ^ab^ | | 348 ± 47.1 ^ab^ | | 26.7 ± 4.1 ^b^ | | 28.2 ± 4.2 ^b^ | 22.7 ± 9.6 ^a^ | 162 ± 117 ^a^ | | 92.3 ± 14.3 ^ab^ | |
| Side channel | | 20 | 3.8 ± 0.7 ^bc^ | 8.2 ± 2.4 ^b^ | 37.1 ± 18.2 | 264 ± 48.4 ^b^ | | 296 ± 55.8 ^b^ | | 29.4 ± 5.7 ^ab^ | | 26.6 ± 6.2 ^b^ | 19 ± 11 ^a^ | 24.1 ± 7.8 ^b^ | | 44.7 ± 14.9 ^b^ | |
| Ortho. Spring | | 21 | 4.4 ± 0.8 ^b^ | 13 ± 3.5 ^b^ | 21.4 ± 9.9 | 306 ± 67.6 ^ab^ | | 332 ± 65.8 ^ab^ | | 40.2 ± 6.3 ^a^ | | 49 ± 13.8 ^a^ | 3.9 ± 1.4 ^b^ | 82.8 ± 39.4 ^a^ | | 105 ± 29.7 ^a^ | |
| Para. Spring | | 17 | 3.7 ± 0.8 ^c^ | 42.9 ± 23.5 ^a^ | 57.2 ± 30.3 | 155 ± 25.9 ^d^ | | 253 ± 36 ^d^ | | 39.6 ± 8.8 ^a^ | | 43.9 ± 10 ^a^ | 10.8 ± 3.4 ^ab^ | 121 ± 55.4 ^a^ | | 114 ± 63.5 ^a^ | |
| Pond | | 28 | 7.2 ± 1 ^a^ | 31 ± 11.2 ^a^ | 34.4 ± 27.3 | 408 ± 47 ^a^ | | 479 ± 62.9 ^a^ | | 33.7 ± 5.2 ^a^ | | 39.5 ± 7.9 ^ab^ | 5.8 ± 2.1 ^b^ | 145 ± 41.4 ^a^ | | 866 ± 730 ^a^ | |
|  | |  |  |  |  |  | |  | |  | |  |  |  | |  | |
|  | *[DOC] = Dissolved Organic Carbon; [DON] = Dissolved Organic Nitrogen; [TN] = Total Nitrogen; [SRP] Soluble Reactive Phosphorous; [TP] = Total Phosphorous; [N:P] = molar ratio for (NH4 + NO3)/SRP; [Chl-a] = Chlorophyll-a abundance; [AFDM] = Ash Free Dry Mass.* | | | | | | | | | | | | | | | | |

**Figure S2 | Flow predictability and floodplain complexity.** Flow predictability (i.e., Colwell’s M-index) versus a) floodplain habitat diversity, and b) environmental heterogeneity in biofilm habitats. Habitat diversity was estimated by calculating Shannon-Wiener diversity index on count data for each habitat type and floodplain (n=265). Environmental heterogeneity among epilithic biofilm habitats was estimated as the Coefficient of Variation (CV) of the overall distance to centroid from the PCA analysis (n=126).


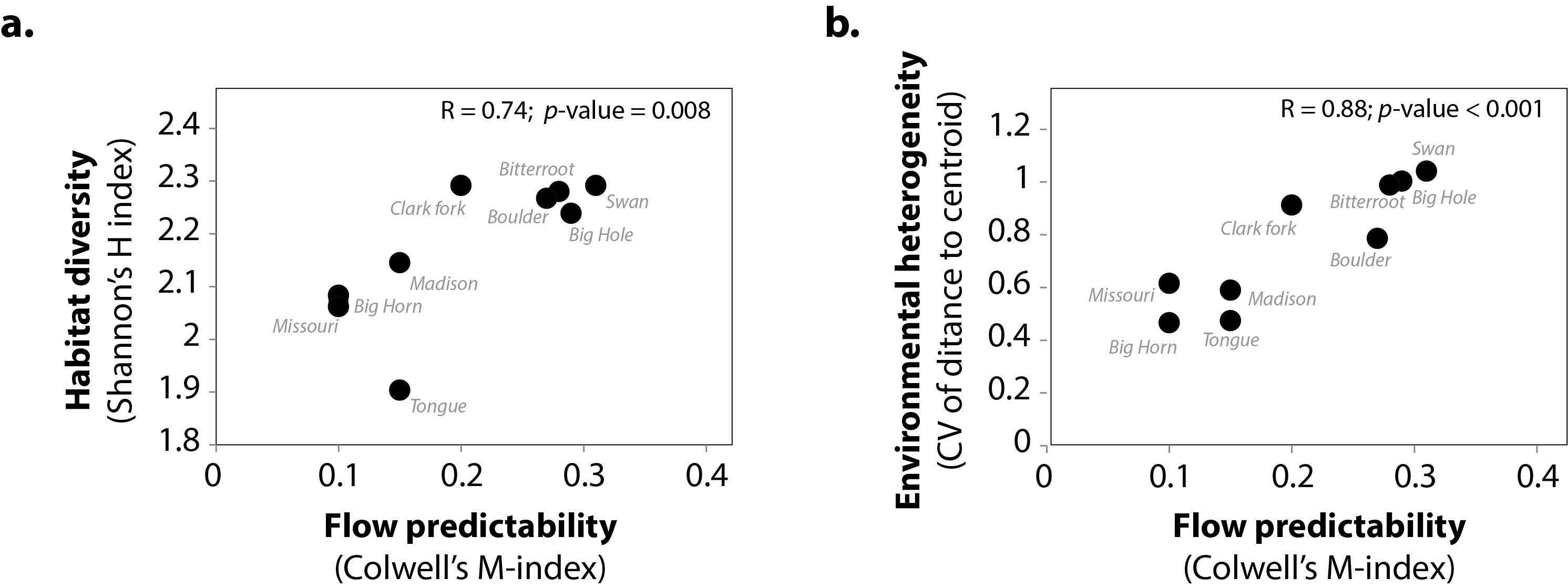


| **Table S2 \| Multi-scale bacterial diversity.** Mean values and standard deviation of alpha (as Shannon’s H index) and beta (as Sørensen index) diversity for each biome, floodplain, zone, and habitat type. Partitions of beta diversity (species turnover and nestedness) are also provided. Within a column, unique superscripts denote significant (*i.e.,* P < 0.05) differneces among floodplains | | | | | |
| --- | --- | --- | --- | --- | --- |
|  | **α-diversity** | **β-diversity** | **β_turnover_** | **β_nestedness_** | **n** |
| **Biome** |  |  |  |  |  |
| Grassland | 3.4 ± 0.5 | 0.59 ± 0.10 | 0.52 ± 0.11 | 0.06 ± 0.10 | 25 |
| Montane | 3.2 ± 0.4 | 0.56 ± 0.15 | 0.55 ± 0.15 | 0.01 ± 0.02 | 91 |
|  |  |  |  |  |  |
| **Floodplain** |  |  |  |  |  |
| Boulder | 3.1 ± 0.2^ab^ | 0.31 ± 0.03^ab^ | 0.26 ± 0.04 | 0.05 ± 0.03 | 9 |
| Clark fork | 3.3 ± 0.3^bc^ | 0.30 ± 0.03^a^ | 0.24 ± 0.05 | 0.06 ± 0.06 | 13 |
| Big Hole | 3.1 ± 0.6 ^ab^ | 0.34 ± 0.05^cb^ | 0.26 ± 0.06 | 0.08 ± 0.06 | 15 |
| Swan | 3.4 ± 0.9^bc^ | 0.36 ± 0.10^c^ | 0.24 ± 0.05 | 0.13 ± 0.12 | 22 |
| Bitterroot | 3.0 ± 0.3 ^a^ | 0.32 ± 0.05^ab^ | 0.26 ± 04 | 0.06 ± 0.05 | 16 |
| Tongue | 4.1 ± 0.3^d^ | 0.30 ± 0.06^a^ | 0.22 ± 0.04 | 0.07 ± 0.06 | 11 |
| Madison | 3.3 ± 0.4^bc^ | 0.34 ± 0.06^cb^ | 0.24 ± 0.05 | 0.10 ± 0.08 | 16 |
| Big Horn | 3.5 ± 0.4^c^ | 0.35 ± 0.02^c^ | 0.29 ± 0.05 | 0.06 ± 0.04 | 5 |
| Missouri | 2.8 ± 0.7 ^a^ | 0.39 ± 0.05^d^ | 0.30 ± 0.05 | 0.09 ± 0.06 | 9 |
|  |  |  |  |  |  |
| **Zone** |  |  |  |  |  |
| Main-channel | 3.2 ± 0.7 | 0.62 ± 0.15 | 0.61 ± 0.15 | 0.01 ± 0.02 | 64 |
| Off-channel | 3.4 ± 0.6 | 0.54 ± 0.11 | 0.50 ± 0.11 | 0.04 ± 0.07 | 52 |
|  |  |  |  |  |  |
| **Habitat** |  |  |  |  |  |
| Riffle | 3.0 ± 0.7 | 0.64 ± 0.17 | 0.64 ± 0.17 | 0.00 ± 0.01 | 22 |
| Run | 3.3 ± 0.5 | 0.59 ± 0.13 | 0.58 ± 0.13 | 0.00 ± 0.01 | 20 |
| Confluence | 3.0 ± 0.6 | 0.65 ± 0.15 | 0.63 ± 0.13 | 0.02 ± 0.05 | 12 |
| Shoreline | 3.5 ± 0.6 | 0.54 ± 0.13 | 0.53 ± 0.13 | 0.01 ± 0.00 | 19 |
| Backwater | 3.5 ± 1.1 | 0.47 ± 0.21 | 0.32 ± 0.08 | 0.16 ± 0.14 | 4 |
| Side channel | 3.3 ± 0.5 | 0.55 ± 0.11 | 0.55 ± 0.11 | 0.00 ± 0.01 | 11 |
| Ortho. Spring | 3.5 ± 0.4 | 0.59 ± 0.09 | 0.51 ± 0.08 | 0.08 ± 0.07 | 9 |
| Para. Spring | 3.3 ± 0.9 | 0.47 ± 0.07 | 0.44 ± 0.08 | 0.03 ± 0.02 | 9 |
| Pond | 3.7 ± 0.6 | 0.52 ± 0.08 | 0.47 ± 0.08 | 0.05 ± 0.04 | 10 |
|  |  |  |  |  |  |


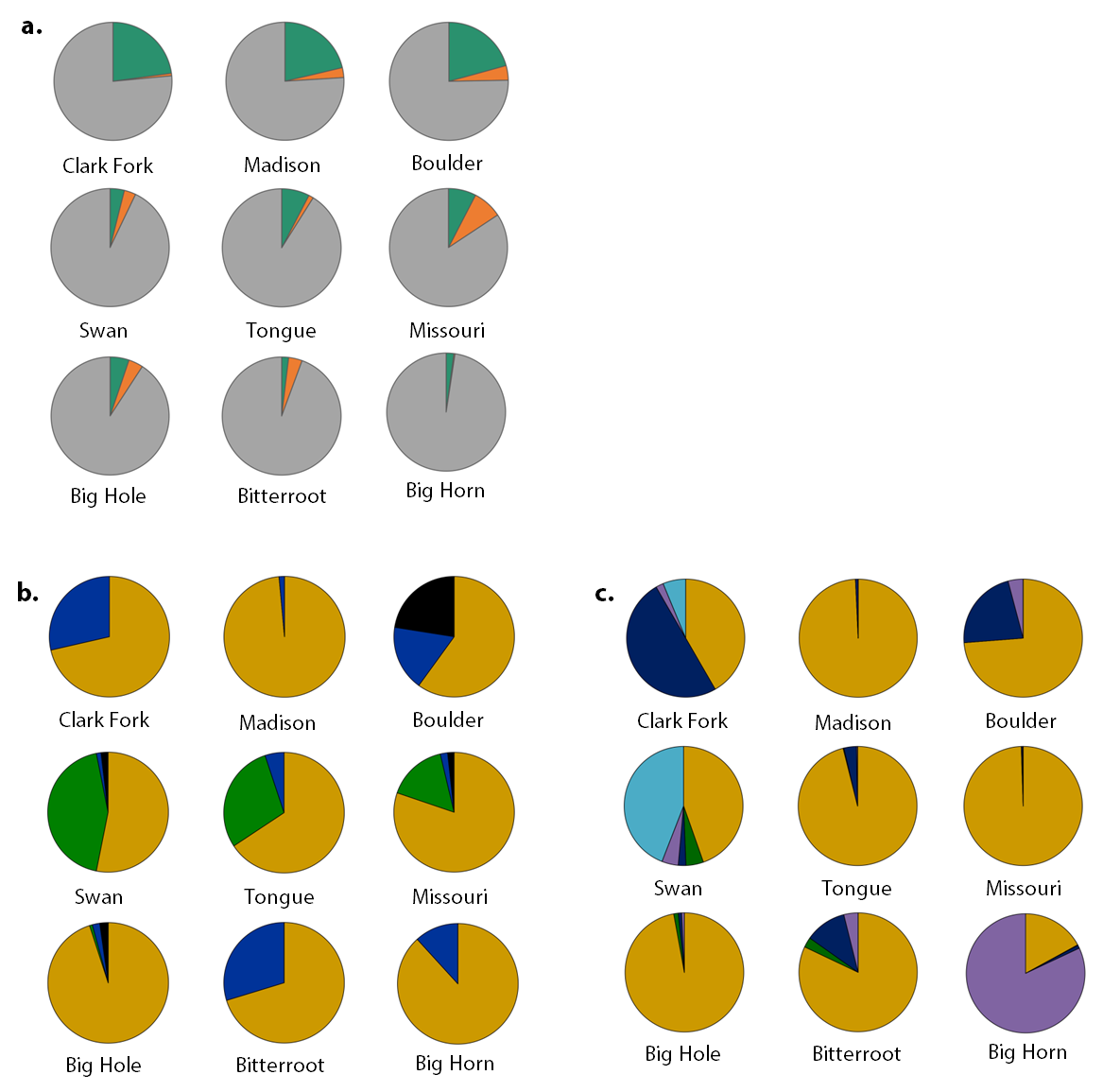


**Figure S3 | Abundance of photosynthetic microorganisms in riverine biofilms.**

**a)** Relative abundance of phototrophs: Cyanobacteria (teal), Eukaryotic algae (orange), and other reads (gray)

**b)** Relative abundances of eukaryotic algae: Stramenopiles (gold), Streptophytes (green), Chlorophytes (blue), and other reads (black)

**c)** Relative abundances of stramenopile algae, Diatoms (gold), chrysophytes (green), eustigmatophytes (dark blue), xanthophytes (lavender), and phaeophytes (light blue)

| **Table S3 \| Pairwise comparisons of bacterial community composition.** ANOSIM results for each pairwise comparison among levels at biome, sub-biome, floodplain, zone, and habitat scale. Sub-biome distinction was revealed by unconstratined ordination via NMDS (see text). The ANOSIM statistic (R) is shown for each comparison. Values of R close to ‘1’ suggest dissimilarity between groups and values approaching ‘0’ suggest similar distribution of high and low ranks within and between groups. All comparisons were significant (*p*-values <0.01) except for those among habitats. | | | | | | | | | |
| --- | --- | --- | --- | --- | --- | --- | --- | --- | --- |
| **Biome** | montane *vs.* grassland | | 0.279 |  |  |  |  |  |  |
|  |  |  |  |  |  |  |  |  |  |
| **Sub-biome** | montane (N-rich) *vs.* grassland | | | 0.215 |  |  |  |  |  |
|  | montane (N-limited *vs.* N-rich) | | | 0.543 |  |  |  |  |  |
|  | montane (N-limited) *vs.* grassland | | | 0.687 |  |  |  |  |  |
|  |  |  |  |  |  |  |  |  |  |
| **Floodplain** |  | Big Hole | Bighorn | Bitterroot | Boulder | Clark Fork | Madison | Missouri | Swan |
|  | Bighorn | 0.736 | - | - | - | - | - | - | - |
|  | Bitterroot | 0.889 | 0.891 | - | - | - | - | - | - |
|  | Boulder | 0.855 | 0.931 | 0.573 | - | - | - | - | - |
|  | Clark Fork | 0.839 | 0.947 | 0.721 | 0.713 | - | - | - | - |
|  | Madison | 0.797 | 0.864 | 0.775 | 0.661 | 0.357 | - | - | - |
|  | Missouri | 0.687 | 0.286 | 0.76 | 0.557 | 0.708 | 0.663 | - | - |
|  | Swan | 0.163 | 0.358 | 0.411 | 0.371 | 0.404 | 0.435 | 0.552 | - |
|  | Tongue | 0.646 | 0.585 | 0.882 | 0.855 | 0.821 | 0.787 | 0.522 | 0.192 |
|  |  |  |  |  |  |  |  |  |  |
| **Zone** | main-channel *vs.* off-channel | | | 0.051 |  |  |  |  |  |
|  |  |  |  |  |  |  |  |  |  |
| **Habitat** | 0.021 | *all pairwise comparisons are non-significant* | | | | |  |  |  |
|  |  |  |  |  |  |  |  |  |  |

| **Table S4 \| Redundancy analyses. a)** Results of RDA with forward-selection of measured biogeochemical variables explaining patterns of biofilm community structure at the habitat scale across all floodplains (n=126). **b)** Results of partial redundancy analysis showing the individual and shared fraction of variation explained by geographic and environmental components. Adjusted-R^2^ values are used to indicate specific contribution of selected variables in *a)* and variance partitions for each component in *b).* | | | |
| --- | --- | --- | --- |
| **a. Selected variables** | **F** | ***p-value*** | **Adj-R^2^** |
| Dissolved Organic Carbon | 7.538 | 0.002 | 0.053 |
| Soluble Reactive Phosphorous | 8.789 | 0.002 | 0.061 |
| Total Nitrogen | 2.369 | 0.024 | 0.011 |
| molar N:P ratio | 2.091 | 0.032 | 0.008 |
| Full model | 5.424 | 0.001 | 0.151 |
|  |  |  |  |
| **b. Variance partitions** | **F** | ***p-value*** | **Adj-R^2^** |
| Environmental component | 5.846 | 0.001 | 0.070 |
| Geographic component | 6.601 | 0.001 | 0.068 |
| Shared variation |  |  | 0.085 |
| Residual variation |  |  | 0.776 |
|  |  |  |  |

| **Table S5 \| Keystone taxa.** List of the keystone taxa identified for each biofilm co-occurrence network. Values for each of the three parameters used for keystone identification are shown. Phyla, relative abundance (as number of reads), zone allocation (as ‘0’ being exclusive of off-channel zones and ‘1’ exclusive of main-channel zones), and average contribution to β-diversity in each riverine floodplain are also indicated. | | | | | | | |
| --- | --- | --- | --- | --- | --- | --- | --- |
| **Taxonomy (Family)** | **Node Degree** | **CC** | **BC** | **Phyla** | **Relative abundance** | **Zonation** | **Contribution β-diversity** |
| *Montane N-limited* |  |  |  |  |  |  |  |
| Cytophagaceae | 49 | 0.469 | 0.087 | Bacteroidetes | 20538 | 0.43 | 0.016 |
| Cyclobacteriaceae | 25 | 0.423 | 0.075 | Bacteroidetes | 1109 | 0.18 | 0.004 |
| PeM15_fa | 49 | 0.468 | 0.070 | Actinobacteria | 2591 | 0.48 | 0.003 |
| SC-I-84_fa | 23 | 0.385 | 0.060 | Proteobacteria | 803 | 0.33 | 0.002 |
| NS11-12_ group | 24 | 0.424 | 0.059 | Bacteroidetes | 181 | 0.25 | 0 |
| FamilyI | 35 | 0.435 | 0.057 | Cyanobacteria | 4368 | 0.53 | 0.007 |
| Microbacteriaceae | 40 | 0.455 | 0.055 | Actinobacteria | 1351 | 0.48 | 0.003 |
| Saprospiraceae | 35 | 0.432 | 0.050 | Proteobacteria | 14168 | 0.44 | 0.02 |
| Acidimicrobiales_Incertae_Sedis | 39 | 0.434 | 0.047 | unknown | 476 | 0.53 | 0.001 |
| MNG7 | 37 | 0.436 | 0.044 | Proteobacteria | 6805 | 0.51 | 0.007 |
| HTA4_fa | 28 | 0.421 | 0.041 | Verrucomicrobia | 771 | 0.44 | 0.001 |
| Planctomycetaceae | 30 | 0.423 | 0.040 | Planctomycetes | 23712 | 0.53 | 0.017 |
| OPB56 | 18 | 0.385 | 0.040 | Chlorobi | 247 | 0.25 | 0.001 |
| Rhodospirillales_Incertae_Sedis | 23 | 0.387 | 0.036 | Parcubacteria | 462 | 0.45 | 0.001 |
| Xanthobacteraceae | 29 | 0.421 | 0.034 | Proteobacteria | 634 | 0.51 | 0.001 |
| alphaI_cluster | 20 | 0.390 | 0.034 | Bacteroidetes | 689 | 0.29 | 0.002 |
| Rikenellaceae | 26 | 0.405 | 0.033 | unknown | 562 | 0.45 | 0.003 |
| Parachlamydiaceae | 22 | 0.392 | 0.032 | Planctomycetes | 626 | 0.56 | 0.001 |
| Legionellaceae | 31 | 0.405 | 0.030 | Proteobacteria | 926 | 0.51 | 0.002 |
| Bacteroidetes_fa | 24 | 0.399 | 0.029 | Bacteroidetes | 123 | 0.53 | 0 |
| Hyphomicrobiaceae | 18 | 0.381 | 0.027 | Proteobacteria | 5822 | 0.61 | 0.008 |
| Archangiaceae | 14 | 0.387 | 0.025 | Bacteroidetes | 352 | 0.53 | 0.001 |
| Hyphomonadaceae | 28 | 0.410 | 0.023 | Proteobacteria | 3983 | 0.41 | 0.004 |
|  |  |  |  |  |  |  |  |
| *Montane N-rich* |  |  |  |  |  |  |  |
| Solibacteraceae_(Subgroup_3) | 61 | 0.455 | 0.049 | Proteobacteria | 948 | 0.45 | 0 |
| M05-Pitesti | 58 | 0.428 | 0.036 | Nitrospirae | 135 | 0.61 | 0 |
| Methylobacteriaceae | 53 | 0.425 | 0.035 | Armatimonadetes | 655 | 0.19 | 0 |
| ABS-19 | 61 | 0.433 | 0.034 | Acidobacteria | 55 | 0.29 | 0 |
| Caldilineaceae | 79 | 0.448 | 0.028 | Cyanobacteria | 465 | 0.35 | 0 |
| Subgroup_17_fa | 101 | 0.482 | 0.025 | Chlamydiae | 913 | 0.40 | 0 |
| Xanthomonadales_Incertae_Sedis | 85 | 0.476 | 0.025 | Proteobacteria | 2220 | 0.34 | 0 |
| Tepidisphaeraceae | 103 | 0.484 | 0.025 | Proteobacteria | 952 | 0.43 | 0 |
| Planctomycetaceae | 98 | 0.485 | 0.024 | Planctomycetes | 6479 | 0.44 | 0.01 |
| KD4-96_fa | 96 | 0.470 | 0.022 | Proteobacteria | 1507 | 0.50 | 0 |
| uncultured | 96 | 0.479 | 0.021 | Proteobacteria | 800 | 0.37 | 0 |
| Xanthobacteraceae | 80 | 0.439 | 0.020 | Proteobacteria | 1158 | 0.55 | 0 |
| Chthoniobacteraceae | 92 | 0.483 | 0.020 | Chloroflexi | 1712 | 0.43 | 0 |
| Sporichthyaceae | 93 | 0.464 | 0.019 | Proteobacteria | 1019 | 0.40 | 0 |
| Fimbriimonadaceae | 76 | 0.445 | 0.017 | Firmicutes | 821 | 0.43 | 0 |
| Nitrosomonadaceae | 72 | 0.457 | 0.017 | Proteobacteria | 3325 | 0.30 | 0.01 |
| Elev-16S-1332 | 56 | 0.424 | 0.016 | Actinobacteria | 203 | 0.54 | 0 |
| Subgroup_6_fa | 97 | 0.470 | 0.016 | Actinobacteria | 2613 | 0.51 | 0.01 |
| Subgroup_22_fa | 75 | 0.435 | 0.016 | Acidobacteria | 162 | 0.47 | 0 |
| Roseiflexaceae | 61 | 0.440 | 0.015 | Actinobacteria | 582 | 0.29 | 0 |
| uncultured | 77 | 0.446 | 0.014 | Cyanobacteria | 702 | 0.56 | 0 |
| Phycisphaeraceae | 84 | 0.465 | 0.014 | Actinobacteria | 837 | 0.35 | 0 |
| Latescibacteria_fa | 94 | 0.466 | 0.014 | Latescibacteria | 335 | 0.42 | 0 |
| OM190_fa | 91 | 0.472 | 0.014 | Planctomycetes | 212 | 0.31 | 0 |
| JG35-K1-AG5 | 56 | 0.418 | 0.013 | Proteobacteria | 1041 | 0.55 | 0 |
| Blastocatellaceae_(Subgroup_4) | 98 | 0.478 | 0.012 | Proteobacteria | 1983 | 0.48 | 0 |
| DEV007 | 71 | 0.439 | 0.012 | Proteobacteria | 159 | 0.34 | 0 |
| A0839 | 71 | 0.455 | 0.012 | Verrucomicrobia | 898 | 0.34 | 0 |
| B1-7BS_fa | 83 | 0.456 | 0.012 | Proteobacteria | 122 | 0.35 | 0 |
| Hydrogenedentes_fa | 85 | 0.460 | 0.011 | Hydrogenedentes | 58 | 0.47 | 0 |
| Rhodobiaceae | 78 | 0.444 | 0.011 | Proteobacteria | 315 | 0.50 | 0 |
| TK10_fa | 61 | 0.425 | 0.011 | Chloroflexi | 90 | 0.53 | 0 |
|  |  |  |  |  |  |  |  |
| *Grassland* |  |  |  |  |  |  |  |
| Saccharibacteria_fa | 55 | 0.457 | 0.034 | Proteobacteria | 462 | 0.71 | 0.001 |
| Micromonosporaceae | 93 | 0.493 | 0.032 | SR1_(Absconditabacteria) | 1327 | 0.88 | 0.005 |
| Cyanobacteria_fa | 49 | 0.435 | 0.027 | Cyanobacteria | 391 | 0.41 | 0.004 |
| Oscillochloridaceae | 65 | 0.464 | 0.026 | Chloroflexi | 133 | 0.54 | 0.001 |
| Hydrogenedentes_fa | 71 | 0.466 | 0.026 | Hydrogenedentes | 53 | 0.56 | 0 |
| Saprospiraceae | 99 | 0.504 | 0.025 | Proteobacteria | 2504 | 0.62 | 0.009 |
| Desulfomicrobiaceae | 57 | 0.439 | 0.024 | Proteobacteria | 42 | 0.70 | 0 |
| uncultured | 77 | 0.474 | 0.023 | Cyanobacteria | 514 | 0.73 | 0.001 |
| JG30-KF-CM45_fa | 84 | 0.479 | 0.022 | Bacteroidetes | 718 | 0.75 | 0.002 |
| Desulfobacteraceae | 91 | 0.483 | 0.021 | Proteobacteria | 279 | 0.63 | 0.001 |
| MB-A2-108_fa | 83 | 0.469 | 0.020 | Proteobacteria | 209 | 0.83 | 0.001 |
| Ardenticatenales_fa | 50 | 0.433 | 0.019 | Bacteroidetes | 88 | 0.76 | 0.001 |
| Solibacteraceae_(Subgroup_3) | 75 | 0.463 | 0.018 | Proteobacteria | 706 | 0.65 | 0.001 |
| Chromatiaceae | 70 | 0.463 | 0.017 | Proteobacteria | 262 | 0.58 | 0.001 |
| Sva0996_marine_group | 77 | 0.464 | 0.017 | Actinobacteria | 110 | 0.55 | 0.001 |
| Intrasporangiaceae | 56 | 0.430 | 0.017 | Proteobacteria | 809 | 0.68 | 0.003 |
| Lachnospiraceae | 73 | 0.463 | 0.015 | Firmicutes | 332 | 0.78 | 0.004 |
| Herpetosiphonaceae | 53 | 0.434 | 0.015 | Chloroflexi | 309 | 0.83 | 0.001 |
| Spongiibacteraceae | 52 | 0.450 | 0.014 | Proteobacteria | 80 | 0.55 | 0.001 |
| Kineosporiaceae | 87 | 0.478 | 0.014 | Actinobacteria | 205 | 0.67 | 0.002 |
| Phycisphaeraceae | 57 | 0.457 | 0.014 | Actinobacteria | 545 | 0.57 | 0.001 |
| Chthoniobacterales_Incertae_Sedis | 61 | 0.459 | 0.014 | Firmicutes | 165 | 0.59 | 0 |
| OM1_clade | 80 | 0.472 | 0.013 | Firmicutes | 266 | 0.72 | 0.001 |
| Draconibacteriaceae | 51 | 0.446 | 0.013 | Bacteroidetes | 97 | 0.47 | 0.002 |
| Beijerinckiaceae | 60 | 0.449 | 0.012 | Bacteroidetes | 327 | 0.61 | 0.001 |
| Armatimonadaceae | 58 | 0.441 | 0.012 | Proteobacteria | 129 | 0.45 | 0.001 |
| Thermaceae | 43 | 0.430 | 0.011 | Deinococcus-Thermus | 96 | 0.86 | 0 |
| Marinilabiaceae | 56 | 0.438 | 0.011 | Bacteroidetes | 126 | 0.83 | 0.001 |
| Haliangiaceae | 64 | 0.462 | 0.011 | Proteobacteria | 310 | 0.75 | 0.001 |
| uncultured | 63 | 0.458 | 0.011 | Cyanobacteria | 58 | 0.84 | 0 |
| OM190_fa | 82 | 0.469 | 0.011 | Planctomycetes | 362 | 0.53 | 0.001 |
| JG35-K1-AG5 | 42 | 0.420 | 0.011 | Proteobacteria | 666 | 0.88 | 0.006 |
| Parvularculaceae | 51 | 0.435 | 0.011 | Proteobacteria | 41 | 0.71 | 0 |
| Nitrosomonadaceae | 56 | 0.440 | 0.010 | Proteobacteria | 1184 | 0.59 | 0.003 |
| Unknown_Family | 57 | 0.444 | 0.010 | Aenigmarchaeota | 400 | 0.55 | 0.001 |
| I3A_fa | 54 | 0.436 | 0.010 | Proteobacteria | 32 | 0.51 | 0 |
| TK10_fa | 55 | 0.439 | 0.009 | Chloroflexi | 160 | 0.73 | 0.001 |
| Subgroup_17_fa | 75 | 0.462 | 0.009 | Chlamydiae | 480 | 0.87 | 0.001 |
| PHOS-HE36 | 68 | 0.451 | 0.009 | Ignavibacteriae | 84 | 0.65 | 0.001 |
| Halieaceae | 62 | 0.459 | 0.009 | Verrucomicrobia | 1154 | 0.57 | 0.005 |
| Erysipelotrichaceae | 70 | 0.452 | 0.009 | Firmicutes | 115 | 0.67 | 0.001 |
| Rhodospirillales_Incertae_Sedis | 65 | 0.457 | 0.009 | Parcubacteria | 399 | 0.55 | 0 |
| SUP05_cluster | 45 | 0.437 | 0.008 | Proteobacteria | 65 | 0.50 | 0 |
| FamilyII | 68 | 0.457 | 0.008 | Cyanobacteria | 314 | 0.88 | 0.002 |
|  |  |  |  |  |  |  |  |

**Figure S4 | Keystone taxa and bacterial richness.** Relative abundance of taxa identified as keystone species versus sample richness (at family level) for N-limited (blue), N-rich (orange), and grassland (green) floodplains. Note log scale of horizontal axis. Lines represent significant relationships for grassland (R = 0.83) and N-rich montane (R = 0.87) sub-biomes.


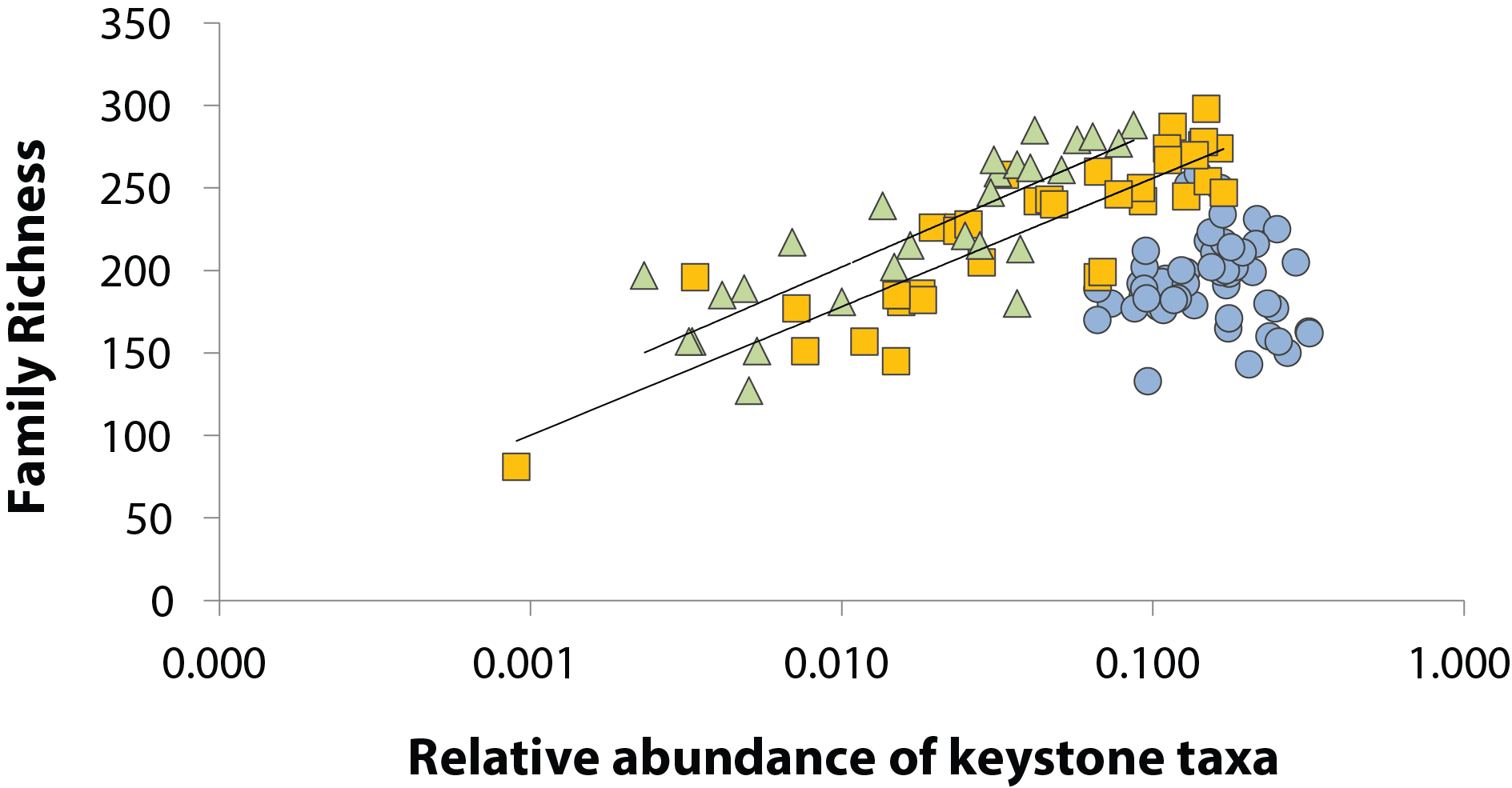


**Figure S5 | Microbial network in N-limited floodplains.** Network graphics *a* and *b* are a replica of Figure 5*a* and *d*, respectively, but with nodes representing taxa of the Cyanobacteria phyla highlighted in blue. Node size is proportional to OTU’s relative abundance (a) and BC values (b). Blue nodes more closely related to heterocystous cyanobacteria (as evidenced by significant regression in number of reads) are labeled as ‘Heterocystous’.

**
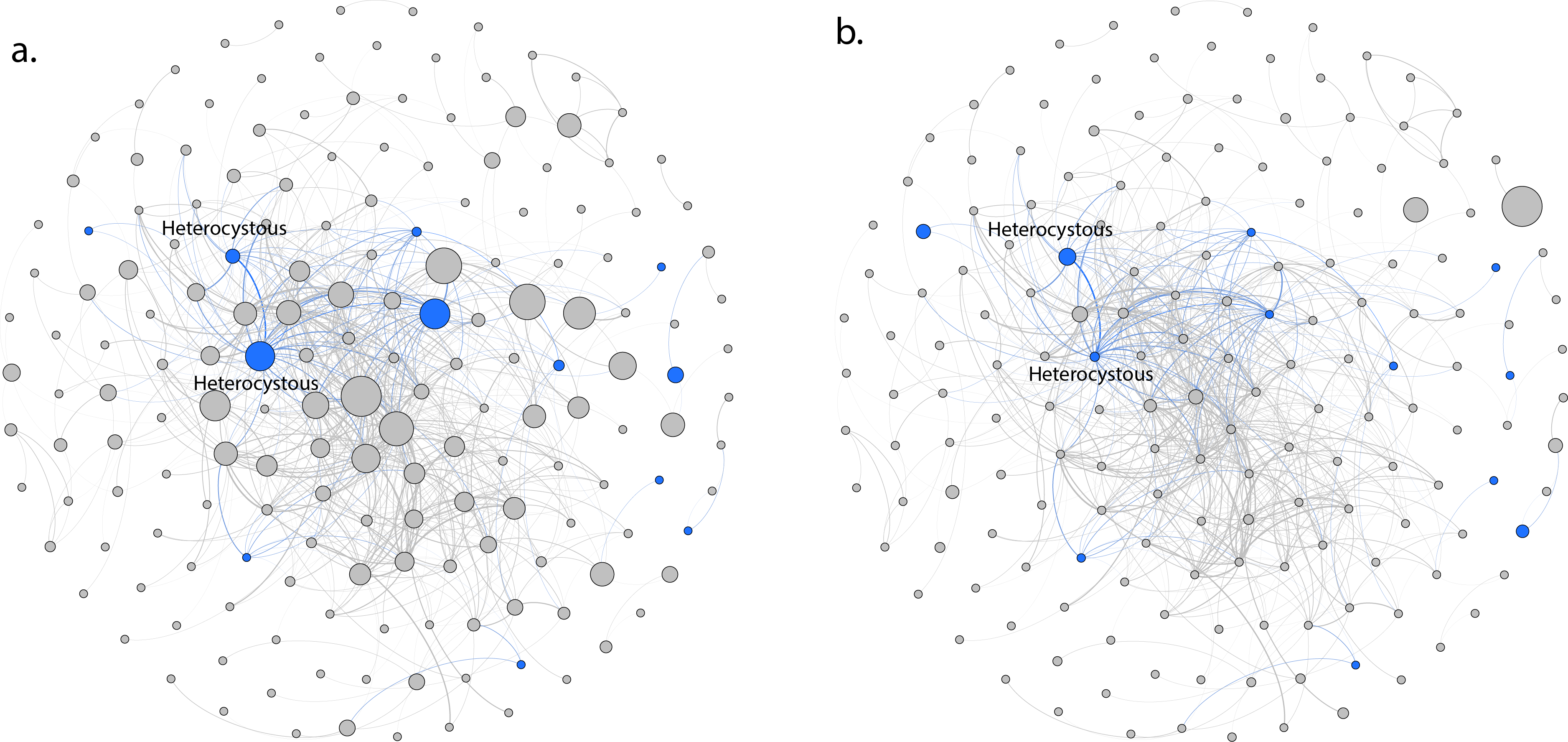
**
